# Phototriggered Desorption of Hydrogen, Ethylene, and Carbon Monoxide from a Cu(I)-Modified Covalent Organic Framework

**DOI:** 10.1021/acs.jpcc.2c03194

**Published:** 2022-08-24

**Authors:** Rachel E. Mow, Lucy J. T. Metzroth, Michael J. Dzara, Glory A. Russell-Parks, Justin C. Johnson, Derek R. Vardon, Svitlana Pylypenko, Shubham Vyas, Thomas Gennett, Wade A. Braunecker

**Affiliations:** † Materials Science Program, Colorado School of Mines, Golden, Colorado 80401, United States; ‡ Department of Chemistry, Colorado School of Mines, Golden, Colorado 80401, United States; § National Renewable Energy Laboratory, Golden, Colorado 80401, United States

## Abstract

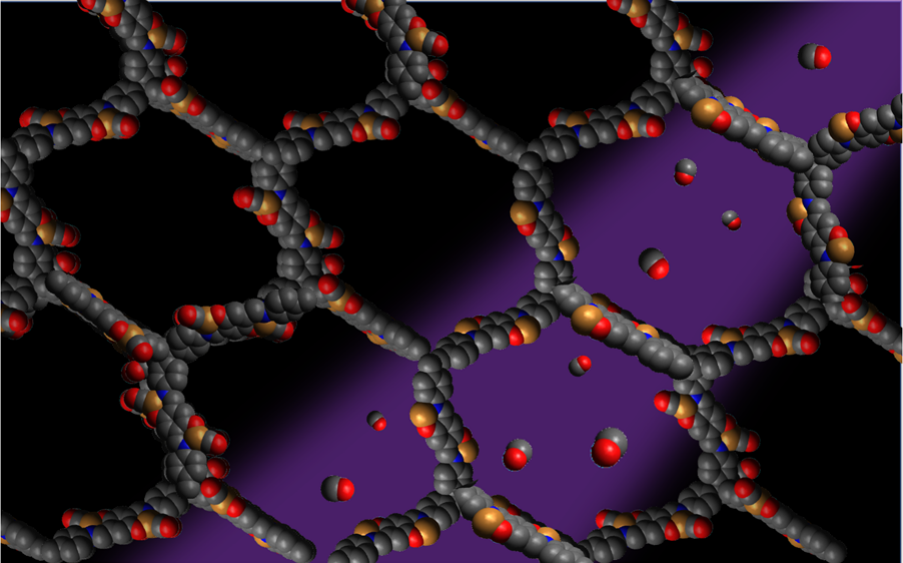

Materials that are capable of adsorbing and desorbing gases near ambient conditions are highly sought after for many applications in gas storage and separations. While the physisorption of typical gases to high surface area covalent organic frameworks (COFs) occurs through relatively weak intermolecular forces, the tunability of framework materials makes them promising candidates for tailoring gas sorption enthalpies. The incorporation of open Cu(I) sites into framework materials is a proven strategy to increase gas uptake closer to ambient conditions for gases that are capable of π-back-bonding with Cu. Here, we report the synthesis of a Cu(I)-loaded COF with subnanometer pores and a three-dimensional network morphology, namely Cu(I)–COF-301. This study focused on the sorption mechanisms of hydrogen, ethylene, and carbon monoxide with this material under ultrahigh vacuum using temperature-programmed desorption and Kissinger analyses of variable ramp rate measurements. All three gases desorb near or above room temperature under these conditions, with activation energies of desorption (*E*_des_) calculated as approximately 29, 57, and 68 kJ/mol, for hydrogen, ethylene, and carbon monoxide, respectively. Despite these strong Cu(I)–gas interactions, this work demonstrated the ability to desorb each gas on-demand below its normal desorption temperature upon irradiation with ultraviolet (UV) light. While thermal imaging experiments indicate that bulk photothermal heating of the COF accounts for some of the photodriven desorption, density functional theory calculations reveal that binding enthalpies are systematically lowered in the COF–hydrogen matrix excited state initiated by UV irradiation, further contributing to gas desorption. This work represents a step toward the development of more practical ambient temperature storage and efficient regeneration of sorbents for applications with hydrogen and π-accepting gases through the use of external photostimuli.

## Introduction

Covalent organic frameworks (COFs) have gained considerable attention due to their crystallinity, high surface areas, composition of lightweight organic elements, and their remarkable tunability.^1−3^ COFs have been implemented in a wide range of applications, including biomedicine and drug delivery,^4−6^ as catalyst supports,^7−9^ in optoelectronics,^10−12^ energy and gas storage applications,^13−15^ and as components for mixed membranes for gas separation.^16^ Gas molecules generally adsorb to high surface area materials via low-energy van der Waals interactions.^17−24^ Physisorbed gases typically require cryogenic temperatures and high pressures to achieve reasonable gravimetric capacities, making long-term gas storage in such materials impractical. However, the tunability of framework materials relative to zeolites and mesoporous oxides affords an opportunity to tailor sorption enthalpies of specific gases. Such tunability can improve both the viability of the materials for storage applications and their selectivity in separations.

The incorporation of open metal sites into framework materials is one way to increase the relative binding strength of different gases that are otherwise difficult to separate due to similarities in size or other physicochemical properties.^25−28^ In particular, open Cu(I) sites have proven effective for selective gas sorption due to the ready interaction between the filled Cu 3d orbitals and guest gas molecules capable of π-back-bonding with Cu(I),^29^ including π-acids such as carbon monoxide (CO) and ethylene (C_2_H_4_). Indeed, several recent studies employing framework-based materials for CO separations have exploited these interactions; for example, π-back-bonding in Cu(I)-loaded MOFs can provide up to 30-fold selectivity for CO over N_2_ sorption at equilibrium, whereas that selectivity was just 2-fold in a comparable Cu(II)-based MOF.^30^ These materials are also highly efficient at separating olefins from paraffins; Cu(I)-loaded MOFs have demonstrated as much as 80-fold selectivity for C_2_H_4_ over C_2_H_6_.^31^ The latter selectivity is particularly noteworthy given that the global annual production of C_2_H_4_ is 170 million tons, and the bulk of that gas is purified from C_2_H_6_ by using energy-intensive cryogenic distillations.^32^ Selective sorbents that can be efficiently regenerated would be of great commercial significance.

In addition to gas separations, Cu(I) frameworks are also being investigated for hydrogen (H_2_) storage applications. The physisorption of H_2_ to most framework materials is considered quite weak with binding enthalpies on the order of 5–9 kJ/mol;^33^ by comparison, most chemisorbed H_2_ in the form of metal hydrides manifest dehydrogenation enthalpies well above 50 kJ/mol.^34^ The U.S. Department of Energy (DOE) considers 15–25 kJ/mol to be closer to ideal for energy efficient H_2_ storage and delivery.^35^ Materials with binding enthalpies in this mesosorption range are known but are quite rare. Recently, a 2D COF was reported with an open Cu(I) site that released H_2_ with a 15 kJ/mol activation energy of desorption (*E*_des_),^36^ and a Cu(I)-loaded metal–organic framework (MOF) was developed with H_2_*E*_des_ near 27 kJ/mol.^37^ Within both of these Cu(I)-loaded framework materials, the abnormally high values of H_2_*E*_des_ were attributed to π-back-bonding, as H_2_ molecules are capable of back-bonding with Cu(I) through unoccupied σ* orbitals. While both of those materials are remarkable in their own right, the development of additional framework-based materials with unique microenvironments for tuning H_2_ mesosorption would serve to advance critical understanding in the field.

The fields of gas storage and separation would also benefit from the exploration of alternative methods for desorption than bulk heating.^38^ Gas-loaded framework materials are typically regenerated through large energy-intensive pressure and/or temperature swings. However, bulk heating can be a slow and energy-intensive process, especially for large volumes of material. Photodriven processes, on the other hand, represent an intriguing option for reducing the time and energy associated with gas desorption. For example, fuel cell-based back-up power systems would greatly benefit from rapid access to photoreleased H_2_, as would many framework regeneration processes where driving off strongly bound gases is costly and time-intensive. While photocatalysis in general has not found widespread adoption on an industrial scale, which has largely been attributed to challenges associated with light penetration and catalyst illumination,^39^ the recent development of techniques to synthesize colloidal, fluidized framework particles^40,41^ holds great potential as a light management strategy for processes at scale.

This report outlines a methodology for postsynthetic incorporation of open Cu(I) sites into a 3D imine-based COF-301. The activated Cu(I)-loaded COF material was found to reversibly bind H_2_ with *E*_des_ values very near the ideal range of enthalpies targeted by the DOE, and with a record high value for any known COF materials. Furthermore, experimental and computational analyses were employed to investigate efficient desorption of H_2_, C_2_H_4_, and CO via reversible photodriven processes.

## Experimental Methods

### General

The tetrakis(4-aminophenyl)methane monomer was purchased from TCI America, and the 2,5-dihydroxyterephthalaldehyde monomer was purchased from AChemBlock. The Cu(II) formate was purchased from Alfa Aesar. Heavy walled glass pressure vessels with PTFE caps were used in the syntheses of all COF materials. A Thorlabs mounted 385 nm LED with a focusing lens were used for all irradiation studies.

### Cu–COF Synthesis

COF-301 was synthesized by dissolving tetrakis(4-aminophenyl)methane and 2,5-dihydroxyterephthalaldehyde in 10 mL of 1,4-dioxane. After the addition of 0.9 mL of acetic acid and 1 mL of deionized water, the mixture was stirred at room temperature (r.t.) for 30 min. A yellow amorphous solid was isolated and purified in 1,4-dioxane to remove any excess monomers. The amorphous solid was combined with 10 mL of 1,4-dioxane, 1 mL of acetic acid, and 5 mL of deionized water in a heavy walled glass pressure vessel with a PTFE cap. The mixture was purged with N_2_ for 15 min and then stirred at 120 °C for 72 h. An orange crystalline solid was isolated and purified by stirring and filtering 3× in 25 mL of 1,4-dioxane and then 3× in 25 mL of acetone over the course of several days. COF-301 was loaded with copper by dissolving 140 mg of Cu(II) formate in methanol and then combining with 100 mg of COF-301. The mixture was stirred at r.t. overnight and yielded a dark brown powder. The Cu-loaded COF was purified by stirring and filtering 3× in 25 mL of methanol for 1 h at r.t. The isolated solid was dried under vacuum (100 mTorr) at 50 °C for 48 h. To convert the Cu(II) formate to open Cu(I) sites, the Cu-loaded COF-301 was activated at 200 °C for several hours under high vacuum (10^–8^ Torr). This activation procedure caused the formate ion to reduce Cu(II) to Cu(I), decomposing into CO_2_ and H_2_ in the process (observed with mass spectrometry) which was pumped away to leave an open Cu(I) binding site.

### Temperature-Programmed Desorption (TPD)

TPD measurements were performed on a calibrated, custom-built system equipped with a Stanford Research Systems RGA 100, capable of measuring *m*/*z* = 1–100 amu. An *m*/*z* range of 1–50 amu was used for each gas in these experiments to increase the data sampling to <3 s/data point. Approximately 2 mg of material was used for each measurement and degassed at 200 °C prior to gas exposure. The samples were dosed at 1.5 bar of the gas of interest (H_2_, C_2_H_4_, or CO) for 10 min at 50 °C. For the H_2_ experiments, the sample was then quenched to 77 K with liquid nitrogen, and the head space was evacuated until the H_2_ signal reached baseline. For the C_2_H_4_ and CO experiments, the head space was evacuated at room temperature for 10 min where baseline signals were reached. A type K thermocouple was used to monitor the temperature, and the samples were heated at a range of ramp rates from 5 to 30 °C/min. A 385 nm LED with an attached focusing lens was used to initiate the phototriggered gas desorption, with power levels ranging from 1 to 300 mW/cm^2^. The H_2_-loaded Cu(I)–COF-301 was immersed in a quartz ice bath during irradiation because H_2_ slowly desorbed from the Cu(I) site at room temperature. The C_2_H_4_- and CO-loaded Cu(I)–COF-301 were irradiated at room temperature. The LED power was controlled by changing the current input; measured irradiances are summarized in Table S1. The sample was agitated during irradiation to expose more material to the light. Experimental parameters were controlled via a LabView interface that is connected to the RGA, heating system, and pressure gauges. The output signal from the mass spectrometer was divided by the total sample mass to get a normalized signal. The baseline pressure before heating was approximately 10^–8^ Torr.

### Transmission Electron Microscopy (TEM)

Scanning transmission electron microscopy (STEM) images and the corresponding energy-dispersive X-ray spectroscopy (EDS) hypermaps were collected by using an FEI Talos F200X operated at 200 kV. Samples were suspended in acetone and dropped onto a 300-mesh gold grid with lacey Formvar/carbon (Ted Pella, 060821). Elemental EDS maps were both collected (acquisition time 5 min) and processed by standard methods using Bruker ESPRIT software.

### Diffuse Reflectance Infrared Fourier Transform Spectroscopy (DRIFTS)

DRIFTS measurements were performed by using a Thermo Scientific Nicolet 6700 with a liquid N_2_ cooled HgCdTe detector and a Harrick Scientific Praying Mantis diffuse reflection accessory. The sample was loaded with CO ex situ and then prepared as a 10 wt % mixture in a KBr matrix inside the glovebox. Background and sample measurements were performed under a He environment.

### X-ray Photoelectron Spectroscopy (XPS)

XPS measurements were performed by using a custom Scienta-Omicrometer HiPP-3 system equipped with an R4000 hemispherical analyzer operating in transmission mode, calibrated to the Au 4f region (83.95 eV) of a sputter-cleaned Au foil. A focused Al Kα X-ray source (1486.6 eV) was operated with a 900 μm spot size at 300 W. The X-ray beam was incident normal to the sample, and emitted photoelectrons were collected at an emission angle of 45° to the direction of the incident X-ray beam. Survey and high-resolution spectra (Cu 2p, N 1s, O 1s, and C 1s) were acquired at pass energies of 500 and 200 eV, respectively, and with slit dimensions of 4.0 × 30 mm^2^, resulting in a resolution of 2 eV for the survey, and a 0.8 × 30 mm^2^ slit size was used, resulting in an estimated energy resolution of approximately 0.59 eV for the core levels. Survey spectra were acquired with a step size of 1 eV, while core levels were acquired with a step size of 0.1 eV. The analysis chamber was maintained at a pressure near or below 5.0 × 10^–8^ mbar, while the analyzer pressure remained below 1 × 10^–9^ mbar. Powder samples were mounted on nonconductive double-sided tape, and a low-energy electron source was operated at 5.0 V with a beam current of 50 μA to compensate for charge accumulation. Binding energy (BE) calibration was performed by adjusting the C 1s core level to 285 eV. Spectral processing was performed with CasaXPS software, where a Shirley background was applied to all core levels.

### X-ray Diffraction (XRD)

XRD measurements were performed on a PANalytical PW3040 X-ray diffractometer using Cu Kα (λ = 1.54 Å) radiation. The scan rate was 2°/min, with a current of 40 mA and a voltage of 45 kV.

### Physisorption Measurements

Brunauer–Emmet–Teller (BET) adsorption isotherms were collected on a Micromeritics ASAP 2020. Each sample was degassed at 200 °C prior to analysis and transferred to the physisorption instrument without air exposure. N_2_ isotherms were collected at 77 K with a 10 s equilibration time. A density functional theory (DFT) slit-pore model was used to extract pore size distributions.

### Thermal Imaging

A FLIR E6-XT series thermal imaging camera was employed with a temperature range of −20 to 550 °C, a thermal sensitivity of 60 mK, a 9 Hz frame rate, and a temperature accuracy of ±2 °C. All measurements were performed inside a dry N_2_-filled box. The emissivity of the COF samples was estimated by calibrating against matte black electrical tape with a known emissivity of 0.95. COF-301 and Cu(I)–COF-301 were estimated to have emissivities of 0.85 and 0.90, respectively.

## Computational Methods

All computational work was performed by using the Gaussian 16 RevC.01 software package.^42^ A representative “linker molecule” structure that shows only the interaction of the main COF forming structure with a respective adsorbate was utilized for the quantum chemical calculations (Figure S23). All ground-state (S_0_) and excited-state (S_1_) geometries were optimized by using the Coulomb attenuating method (CAM)-B3LYP functional which has added long-range orbital–orbital exchange interaction needed to fully describe the interaction of available π electrons in the adsorbate in these systems.^43^ 6-31+G(d) basis sets were applied to all atoms except the metal center, which was treated with 6-311+G(d) basis sets. To compute vertical excitations and excited-state properties, calculations were performed with the time-dependent CAM-B3LYP (TD-DFT)^44,45^ formalism as implemented in Gaussian 16 software. Obtained stationary points were characterized as minima by verifying the absence of any imaginary frequencies. TD-DFT calculations were benchmarked to experimental UV–vis data to confirm proper correlation to the linker molecule (Figure S20). For full accounting of TD-DFT see Tables S3–S12.

The enthalpy of binding, Δ*H*°_bind_, was found from the following expression:
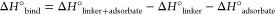
where Δ*H*°_linker+adsorbate_ is the enthalpy of the complex formed between the COF linker molecule and the adsorbate, Δ*H*°_linker_ is the enthalpy of the linker molecule alone, and Δ*H*°_adsorbate_ is the enthalpy of the adsorbate alone.

Potential energy surface scans were performed in the first excited state by using their respective minimum-energy structure. The degree of freedom in these scans utilized the coordinate describing binding of the adsorbate with the COF linker to mimic desorption. The highest energy point on these potential energy surfaces described the upper limit of a transition state where the adsorbate likely detached from the linker molecule (Figures S24–S26). Δ*H*°_bind_ was then determined by using the above equation where the transition state enthalpy was considered equivalent to Δ*H*°_linker_ + Δ*H*°_adsorbate_, giving an upper bound of the excited-state binding enthalpy. Electron difference density plots were obtained by subtracting the ground-state electronic density from the Franck–Condon excited-state density (calculated during TD-DFT), showing the movement of electrons upon excitation for each adsorbate system.

## Results and Discussion

### Cu(I)–COF-301 Synthesis and Characterization

Recently we discovered that Cu can be loaded into a 2D COF at phenol imine docking sites and store appreciable H_2_ near ambient conditions in the material.^36^ Because phenol–imine intramolecular hydrogen bonding is now a commonly employed motif in COF materials to suppress the torsion of neighboring phenyl groups and improve overall crystallinity,^46^ the literature contains a host of promising candidate materials with potential Cu binding sites. Here, we focus our study on a Cu-loaded 3D imine-based COF known in the literature as COF-301. Although a recent theoretical study looking at the H_2_ storage potential of a series of different transition-metal-loaded COFs identified COF-301 as a promising material,^47^ to our knowledge, no experimental work has been published on the H_2_ storage properties of any metal-loaded COF-301 derivatives. Furthermore, while 2D COFs form relatively large channel-like pores with long-range order, 3D COFs tend to have intertwined morphologies with subnanometer size pores and unique microenvironments. The small pores can be advantageous when incorporated into mixed polymeric membranes employed for gas separations as they are less likely to be clogged with polymer.^48^

COF-301 was synthesized following an adapted literature procedure outlined in the Experimental Methods, yielding a bright orange powder. Brunauer–Emmett–Teller (BET) analysis of our material revealed a surface area of 770 m^2^ g^–1^, in line with the literature value for this material.^49^ Characterization of the IR stretching frequencies of the COF product is fully consistent with literature values for COF-301^49,50^ and other imine-based COFs employing this linking chemistry;^51^ for example, well-defined C=N imine stretching appears near 1610 cm^–1^ (Figure S5).

In general, the postsynthetic installation of open metal binding sites suitable for H_2_ storage into any framework material is not trivial, and COF-301 is no exception. Despite the aforementioned promising theoretical studies of COF-301 derivatives for H_2_ storage, literature attempts to load COF-301 with Pd salts were unsuccessful, which was attributed to the small pore size of the COF and relatively large size of the solvated metal species.^24^ However, this work found that COF-301 could be efficiently loaded with Cu(II) formate by modifying our previous procedure.^36^ Cu(II) formate is advantageous over other Cu salts for these purposes as the formate ion can efficiently reduce Cu(II) to Cu(I) upon heating, becoming CO_2_ and H_2_ in the process which can then be pumped away under vacuum to leave an open Cu(I) binding site. After loading COF-301 with Cu(II) formate by stirring in a methanol solution and then filtering and washing, the orange material becomes red-brown. ICP analysis following Cu(II) loading suggested COF-301 uptakes approximately 10 wt % Cu. TEM imaging and elemental mapping confirm the dispersity of Cu in the COF pores (Figure S1).

**Figure 1:**
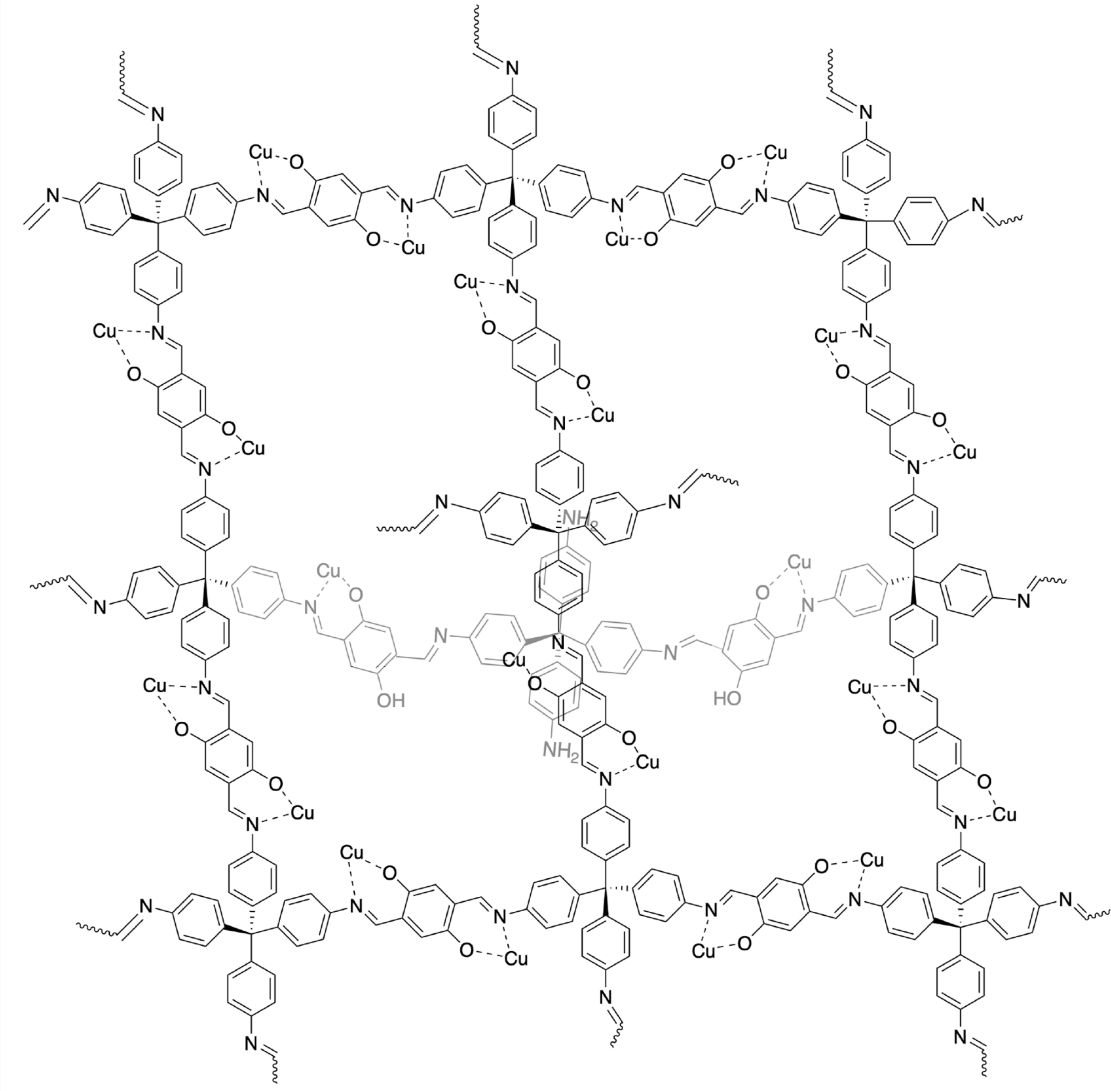
Idealized chemical structure of Cu(I)-COF-301, represented as a single open pore. For clarity, the intertwined pore structure of this 3D COF is not represented.

DRIFTS data of the COF before and after loading with Cu suggest the metal interacts with both the imine functionality and the phenolic OH (Figure S5). We assign the band at υ(1340 cm^–1^) in the parent COF to phenolic C–O stretching based on a literature value of 1344 cm^–1^ for this stretch in a model compound (2,5-bis(phenyliminomethyl)-1,4-benzenediol)^52^ that is representative of a repeating fragment in COF-301. In some other well-characterized Schiff base complexes with Cu^53^ and Co,^54^ phenolic C–O stretching shifts from approximately 1280 to 1320 cm^–1^ upon metal complexation and imine C=N stretching from around 1610 to 1590 cm^–1^, indicative of metal coordination through both phenolic oxygen and imine nitrogen. Here, we see a similar shift in the phenolic C–O band from 1340 to 1360 cm^–1^ with Cu loading. Furthermore, we observe a marked decrease in the broad phenolic O–H stretching between 3200 and 3500 cm^–1^ as well as the disappearance of imine C=N stretching at 1610 cm^–1^, which presumably shifts to overlap with the C=C stretching band near 1590 cm^–1^. The results are thus fully consistent with the formation of a Schiff base complex like the idealized structure depicted in Figure 1; however, it is possible Cu may simultaneously interact with more than one imine site from parallel intertwined layers in this crystalline 3D COF, as has been suggested for other Pd^9^ and Cu^55^ loaded COFs with similar binding sites.

**Figure 2:**
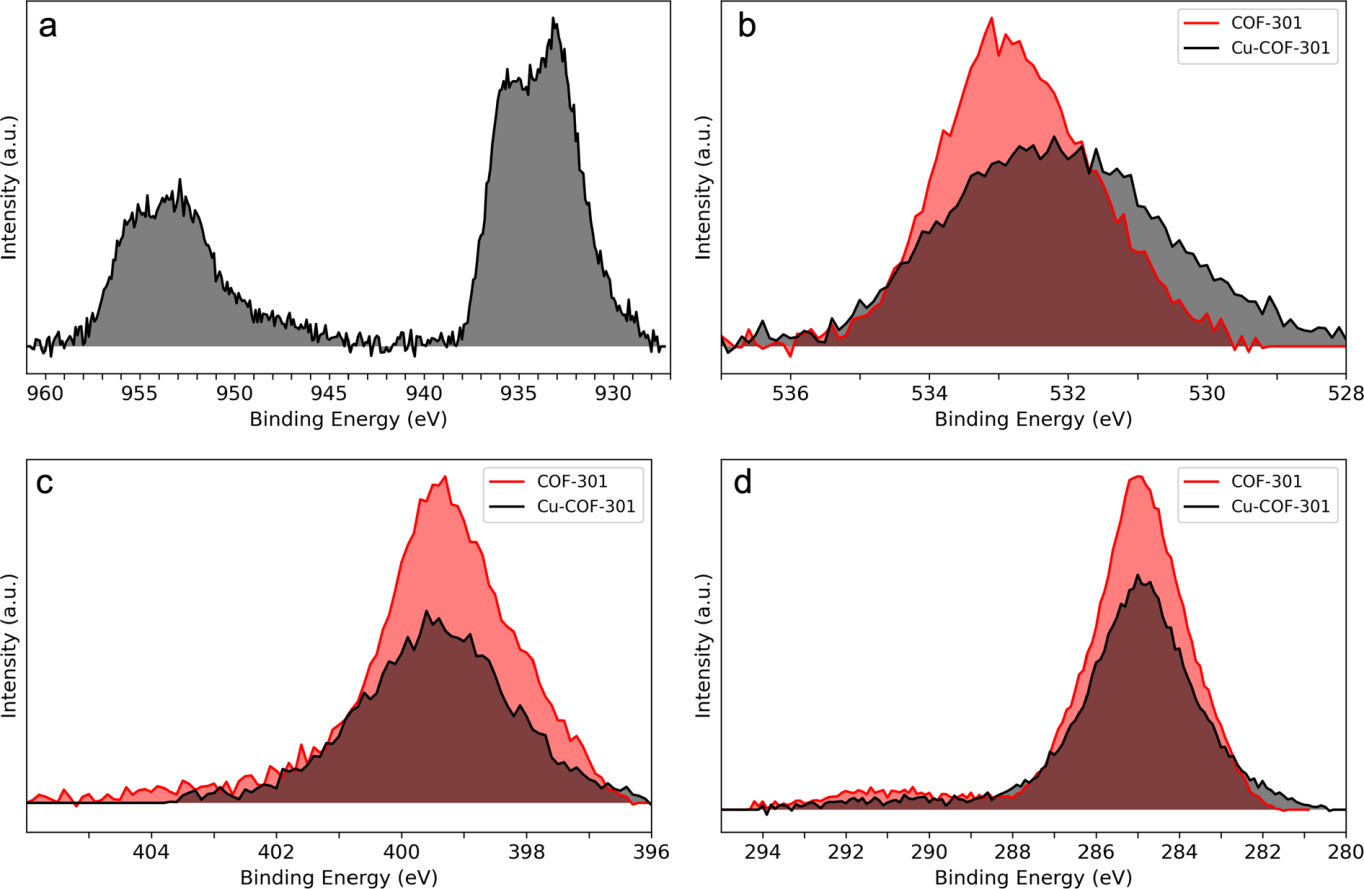
High-resolution XPS spectra for (a) Cu 2p, (b) O 1s, (c) N 1s, and (d) C 1s.

Following activation of Cu(II)–COF-301 at 200 °C (see Experimental Methods), the Cu-loaded COF turns black. XPS was used to supplement the DRIFTS data and probe the nature of Cu binding in these materials as well as to confirm the oxidation state of Cu after activation. XPS survey spectra (Figure S8) confirm the presence of Cu after the loading procedure with characteristic binding energies appearing between 930 and 955 eV (Figure 2a). Core level spectra indicate the O 1s binding energy (Figure 2b) of the phenolic C–O in the parent COF shifts from 533.1 to 532.2 eV when loaded with Cu, consistent with literature values for an analogous Schiff base compound upon Cu complexation (532.4 eV).^56^ The N 1s binding energy also shifts upon complexation (399.3–399.5 eV), and no shift is observed for the C 1s binding energy at 285.0 eV (Figures 2c and 2d, respectively), indicating the Cu interacts with the phenol–imine site in COF-301. The high-resolution XPS Cu 2p spectrum, shown in Figure 2a, indicates the activation procedure was effective at reducing Cu(II) formate. The Cu 2p_3/2_ satellite peaks (940–945 eV) characteristic of the Cu(II) shake-up loss feature notably disappear postactivation (Figure S9), indicating a complete reduction of the Cu(II) species.^57,58^ Although it is difficult to definitively rule out the presence of Cu(0) by using XPS, the observed binding energies and previous XANES experiments performed on a similar material strongly suggest Cu is present as Cu(I).^36^

Finally, powder X-ray diffraction (PXRD) was used to characterize the material before and after Cu loading and activation. The diffraction pattern for the parent COF (Figure S7) was fully consistent with patterns observed in the literature for COF-301,^49,50^ with the strongest peak near 8.4° 2θ. However, unlike 2D COFs with this binding site, where all the original diffraction peaks are retained with metal loading,^9,36,55^ nearly all the diffraction peaks from the parent COF-301 are substantially shifted after Cu(II) loading. Additional but more subtle shifts are observed after activation to Cu(I) (Figure S7). The results are not entirely surprising given that 3D COFs are generally much more flexible than 2D COFs. Indeed, in a recent single-crystal XRD study of the structurally related COF-300, a full 34% reduction in the unit cell volume of the COF-300 crystal was observed when the material was exposed to moisture.^59^ A strong cooperative effect of hydrogen bonds among the guest water molecules bound to the imine functionality of the framework was believed to cause a significant distortion in the overall structure. The presence of Cu guest molecules at imine sites in COF-301 appears to induce its own distortions in the structure. Those results are consistent with a reduction in surface area (Figure S2) and changes observed in the pore size distribution after Cu loading (Figure S3).

### Gas Sorption

Understanding the nature of gas bonding in a framework material is critical to designing highly efficient and selective adsorbents. We began our probe of these interactions at the Cu(I) site in COF-301 with DRIFTS. Because gases such as CO are good σ donors as well as π acceptors, metal bonds with CO can have significant contribution from a M ← CO σ component as well as a M → CO π component. The latter is known as π-back-bonding, where in the case of Cu(I), 3d_π_ electrons donate to the π*orbital of CO.^60^ When values of υ(CO) are lower for M–CO than free CO (the latter occurs at 2143 cm^–1^), π-back-bonding is said to dominate, and indeed this is considered the classical picture for most M–CO species. However, more than 200 M–CO complexes^60^ are known for which υ(CO) is shifted to higher frequencies in the bound state, including numerous Cu(I) complexes, where steric repulsion from other ligands and the nature of the counterions can disproportionately affect the different bonding contributions.

**Figure 3:**
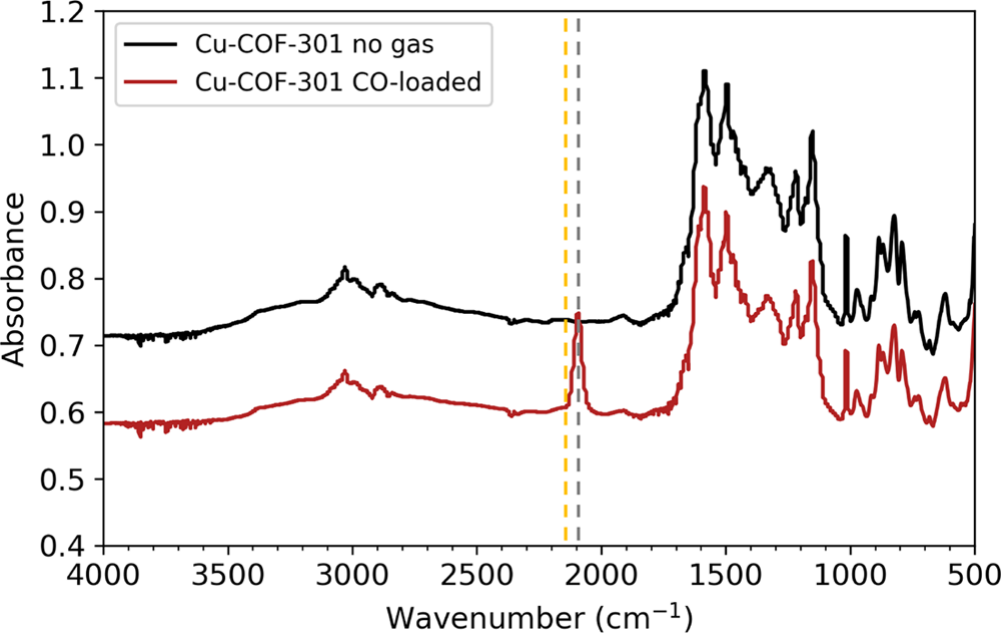
DRIFTS spectra shows CO bound to Cu(I) with a stretching frequency at 2090 cm^–1^ (gray). This stretch was shifted to lower frequencies from free CO (yellow, 2143 cm^–1^), indicative of strong π-back-bonding Cu(I)–CO interactions in these materials.

Here, the Cu(I)–COF-301 complex was dosed with CO ex situ, with subsequent DRIFTS spectra of the complex obtained under a flow of He at 25 °C. As can be seen in Figure 3, bound CO shifts to a lower frequency at 2090 cm^–1^, consistent with strong π-back-bonding at the Cu(I) site and generally consistent with the computationally predicted shift from 2248 to 2212 cm^–1^ for free and Cu(I)-bound CO, respectively. The results suggest Cu(I)–COF-301 might also be selective for other gases that can π-backbond with Cu, i.e., olefins^61−63^ through π*orbitals or H_2_^64−67^ through σ*orbitals. To test this hypothesis, the Cu(I)–COF-301 complex was exposed to ethylene (C_2_H_4_). The bound C_2_H_4_ was more difficult to observe given that all of the vibrational frequencies associated with C_2_H_4_ overlap with those of the COF. While a weak band associated with C=C stretching frequency of adsorbed C_2_H_4_ has been observed between 1535 and 1545 cm^–1^ in a Cu(I)-loaded zeolite^68^ and Cu(I)-containing MOF,^69^ shifting from 1625 cm^–1^ in free C_2_H_4_, we cannot unambiguously assign this stretch in our own material due to the overlap of other strong aromatic and imine stretching frequencies. However, an absorption band associated with CH_2_ scissoring was observed at 1428 cm^–1^ in C_2_H_4_ bound to zeolitic Cu(I),^68^ shifted from 1442 cm^–1^ in free C_2_H_4_. Although the latter band is rather weak, it does appear near 1424 cm^–1^ when the Cu(I)–COF is dosed with C_2_H_4_ (Figure S6). Additional weak bands in the literature complex were observed at 1264 and 930 cm^–1^, assigned to CH_2_ scissoring and wagging, respectively. We observe these bands near 1250 and 930 cm^–1^ as well. Ultimately, the results suggest that stable complexes of Cu(I)–COF-301 can be made at r.t. with both CO and C_2_H_4_. Under the conditions employed for these DRIFTS measurements, however, the complexation of H_2_ was not observed directly.

The interactions of H_2_, C_2_H_4_, and CO with Cu(I)–COF-301 were further probed across a range of temperatures by using temperature-programmed desorption (TPD). Gas desorption is monitored as a function of temperature in TPD; therefore, if a given framework material has multiple binding sites with different heats of adsorption, multiple desorption events can be observed and investigated. For example, we have previously used this technique to quantify adsorption to multiple sites within metal-loaded framework materials.^36^ Here, the analysis for three different gases was conducted by heating the sample with ramp rates ranging from 5 to 30 °C/min, where the peak signal of gas desorption shifts to increasingly higher temperatures with increasing ramp rate. By recording the temperature at the desorption maximum, we can use the following equation to estimate *E*_des_:^70,71^
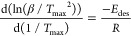
1where β represents the temperature ramp rate, *T*_max_ represents the temperature at the desorption peak, *E*_des_ represents the activation energy of desorption, and *R* is the universal gas constant. Plotting ln(β/*T*_max_^2^) vs 1/*T*_max_ yields a slope representing −*E*_des_/*R*.

**Figure 4:**
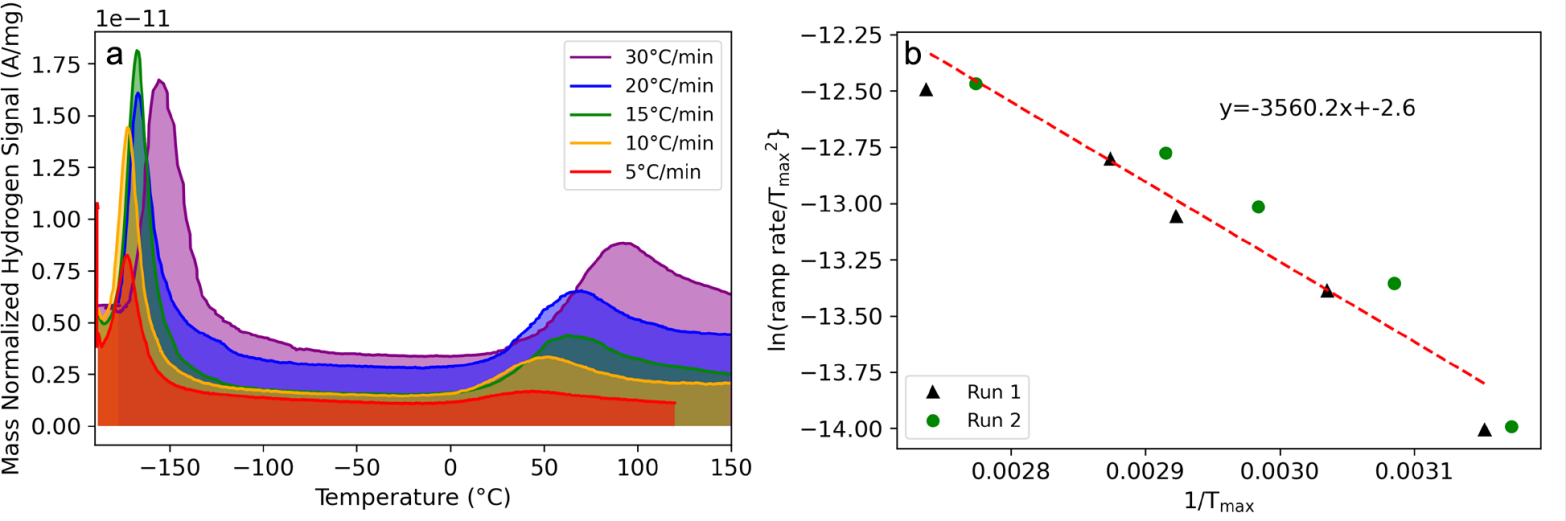
(a) H_2_ variable temperature ramp rate TPD showing physisorbed H_2_ desorbed ∼−160 °C and H_2_ desorbed from the Cu(I) site ∼60 °C under ultrahigh vacuum. (b) Kissinger analysis for two TPD cycles estimated an *E*_des_ of 29 kJ/mol.

Figure 4a shows H_2_ desorption from Cu(I)–COF-301 measured by TPD. The samples were dosed with an overpressure of 1.5 bar of H_2_, and then the headspace was evacuated after the material was quenched with liquid nitrogen. The sample temperature was ramped with rates ranging from 5 to 30 °C/min. Gas desorption was monitored with a mass spectrometer. Two distinct H_2_ desorption peaks were observed, separated by more than 200 °C. The desorption peak near −160 °C corresponds with physisorbed H_2_, while desorption near 60 °C corresponds with H_2_ bound to the Cu(I) site. Note that the latter peak was not observed in the Cu-free COF-301 (Figure S10). Each measurement was duplicated, and the Kissinger method was used to estimate an *E*_des_ of 29 kJ/mol (Figure 4b), the highest reported H_2_*E*_des_ for any known COF-based material^36^ and comparable with state-of-the-art Cu-loaded MOFs.^65,69^ Using the same approach, we estimated *E*_des_ for C_2_H_4_–Cu(I) and CO–Cu(I) as 57 and 68 kJ/mol, respectively (Figures S12 and S14).

We note that the physisorption of gas molecules through weak van der Waals interactions with framework materials generally has a very low activation energy, such that *E*_des_ is a good estimate of binding enthalpy. Indeed, most gas molecules readily physisorb at cryogenic temperatures. However, when these Cu(I)-framework-based materials are dosed with H_2_ at 77 K, the gas physisorbs to the framework but does not adsorb to the Cu(I) site, suggesting the activation energy of adsorption to Cu(I) is not negligible. We observe that when employing an overpressure of 1.5 bar of H_2_, dosing between 25 and 50 °C for 10 min before quenching to 77 K results in the largest quantity of adsorbed H_2_ at the Cu site. However, the general trends in *E*_des_ of these three gases are consistent with trends in the literature adsorption enthalpies and values of *E*_des_.^37,69,72^

Cyclability of the material after exposure to multiple gases was confirmed by performing replicate H_2_ dosing experiments before and after CO and C_2_H_4_ dosing experiments. The results revealed less than 1% reduction in H_2_ uptake at the Cu(I) site across these measurements (Figure S19), indicating adsorption and desorption was fully reversible for all three gases.

### Phototriggered Gas Desorption

As gas-loaded framework materials are typically regenerated through large energy-intensive temperature swings, the bulk heating of large volumes of sorbent materials is an inefficient step in the process. Photodriven desorption processes can in principle provide an alternative method for delivering energy to sorbents rapidly and without bulk heating, reducing the time associated with gas desorption while providing on-demand access to stored gas molecules. A number of different photoprocesses have been proposed and observed to drive the desorption of molecules from organometallic complexes and framework-based materials. For example, the photodesorption of molecular H_2_ from a Ru complex was recently attributed to an optically excited state with reduced Lewis acidity, which effectively weakened Kubas complexation.^73^ Photoinduced metal-to-ligand charge transfer (MLCT) has also been found to induce submicrosecond dissociation of CO, C_2_H_4_, and various acetylene derivatives from Cu(I) and Fe(II) complexes.^61,74−76^ The incorporation of photoswitchable moieties into frameworks that isomerize upon irradiation represents yet another photoinduced mechanism for controlling gas sorption,^77−81^ by inducing localized heating,^82^ physically disrupting the binding of gas molecules in the framework,^83,84^ or by adjusting the electrostatic potential of a specific binding site.^85^

We acknowledge that while a number of fluorescent COFs have recently been developed,^86,87^ the majority of framework-based materials dissipate absorbed electromagnetic radiation through nonradiative decay mechanisms. The latter can induce significant photothermal heating. Indeed, photothermal effects were recently employed to efficiently activate framework-based materials by desorbing moisture and residual solvent.^88^ While it is not always straightforward to disentangle photodriven mechanisms, the irradiation of gas sorbents is nevertheless promising for applications that would benefit from on-demand access to adsorbed molecules.

**Figure 5:**
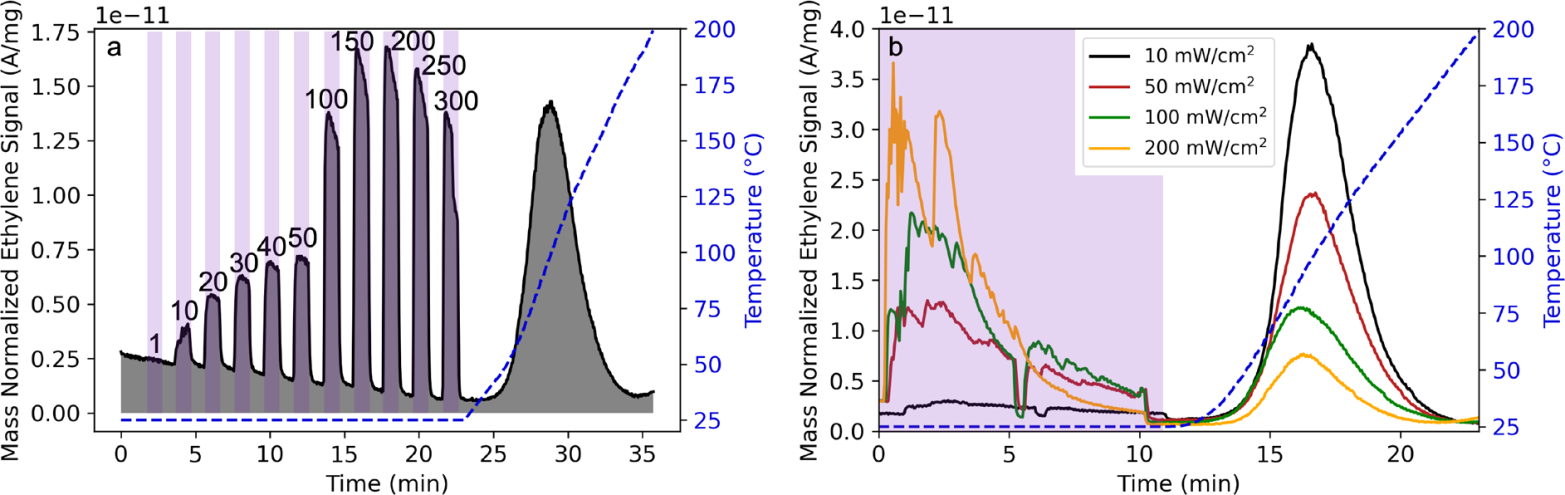
TPD shows on-demand C_2_H_4_ desorption with UV irradiation (purple shaded regions) at r.t. (a) The sample was irradiated for 1 min with powers ranging from 1 to 300 mW/cm^2^ followed by 1 min in the dark. (b) The sample was irradiated for 10 min at four powers, with C_2_H_4_ desorption increasing with irradiance.

Figure 5 illustrates the results of a TPD experiment where Cu(I)–COF-301 was dosed with C_2_H_4_ and then held at a constant 25 °C under vacuum. Under these conditions, C_2_H_4_ desorption is very slow on the time scale of the experiment such that significant desorption does not occur until the temperature is ramped. The sample was then irradiated with 385 nm light (near the local λ_max_ of COF-301, see Figure S20) for 1 min intervals and then kept in the dark for 1 min. Irradiance near the sample was recorded with a light meter and increased with each successive dose of light (between 1 and 300 mW/cm^2^). As can be seen in Figure 5a, C_2_H_4_ desorption occurs nearly instantaneously when the light is switched on and returns to baseline within seconds of the light being switched off. C_2_H_4_ desorption also increased as a function of increasing irradiance (until 150 mW/cm^2^, at which point much of the C_2_H_4_ had already desorbed). A variation of this experiment is illustrated in Figure 5b, where C_2_H_4_-dosed Cu(I)–COF-301 was continuously exposed to 385 nm light for 10 min at a given irradiance before ramping the temperature. The relative fraction of desorbed gas as a function of irradiance was calculated and recorded in Table 1; between 10 and 200 mW/cm^2^, the sample desorbed between 13 and 80% of adsorbed C_2_H_4_, respectively.

**Table 1:** Sample Temperature after 2 min of Irradiation and % C_2_H_4_ and H_2_ Desorbed from the Cu(I) Site after 10 min of Exposure to Different Irradiances

power (mW/cm^2^)	sample temp (°C)	% C_2_H_4_ desorbed with light	% H_2_ desorbed with light
10	25	13	39
50	41	46	50
100	59	66	82
200	85	80	89

Similar experiments were performed with H_2_ (Figures S16 and S17) and CO (Figure S18), with nearly 90% of H_2_ and 70% of CO desorbing from the Cu(I) site with 200 mW/cm^2^ irradiance at 385 nm. The remaining gas was thermally released upon heating after irradiation. The incomplete photodesorption can be explained by poor light penetration deep into the UV-absorbing framework. Replicate variable temperature ramp rate experiments were performed before and after the photodesorption experiments. No measurable change was observed in XRD or the sorption properties of Cu(I)–COF-301 after UV irradiation over the course of the experiments in Figure 5b, indicating irradiation did not permanently alter the structure or properties of the COF.

Thermal imaging experiments conducted on the COF-301 and Cu(I)–COF-301 powders under an inert atmosphere (see Experimental Methods for details) indicate that bulk photothermal heating of the Cu(I)–COF-301 can account for much of the phototriggered gas desorption. The sample temperature was highly dependent on the presence or absence of Cu as well as the irradiance and wavelength employed (Tables S1 and S2). For example, the thermal imaging camera recorded Cu(I)–COF-301 surface temperatures of 97 °C after 30 s of exposure to an irradiance of 225 mW/cm^2^ at 385 nm, while the temperature of the base COF-301 reached 66 °C under these conditions. With 320 mW/cm^2^ of 625 nm light, Cu(I)–COF-301 heated to a similar 95 °C, but the base COF-301 only warmed to 39 °C. These results are not surprising given that the base COF absorbs strongly at 385 nm and very weakly at 625 nm, whereas the black Cu(I)–COF-301 absorbs strongly throughout the visible region. Because this material (like most frameworks) is not fluorescent, the absorbed electromagnetic radiation must dissipate through nonradiative decay, which can contribute to localized heating. However, we note that C_2_H_4_ also rapidly desorbed from the Cu(I) site upon exposure to low irradiances (e.g., 10 mW/cm^2^), where photothermal heating was negligible. To further investigate this phenomenon and probe potential mechanisms involved in gas adsorption and desorption, supplemental computational analyses were conducted.

### Computational Modeling of Cu(I)–Gas Interactions

DFT calculations were first employed to explore the nature of H_2_, C_2_H_4_, and CO interactions with Cu(I)–COF-301 on a model Schiff base compound representative of the Cu(I) binding site in Cu(I)–COF-301 (Figure S23).^89^ The computationally estimated binding enthalpies followed the same trends as the experimentally estimated *E*_des_: CO bound the strongest, followed by C_2_H_4_ and then H_2_ (Table 2). The optimized geometry of the ground state (S_0_) with adsorbed H_2_ shows that H_2_ adsorbed side-on and in-plane with respect to the Cu(I) site. The lengthening of the H–H bond from 0.74 to 0.81 Å and the side-on interaction confirms the presence of a dihydrogen Kubas complex^64^ and is consistent with previously observed bond lengthening in Cu(I)–H_2_ complexes.^37,69^ Bond lengthening was similarly observed when CO and C_2_H_4_ interact with the Cu(I) site, increasing from 1.13 to 1.14 Å and 1.32 to 1.37 Å between the C–O and C–C bonds, respectively.^69,90^ This bond lengthening indicates π-back-bonding is a significant contributor to the strong Cu(I)–gas interactions, consistent with IR spectra obtained both experimentally (Figure 3) and computationally (Figure S4).

**Table 2:** Experimentally Estimated *E*_des_ and Computationally Estimated Binding Enthalpies of H_2_, C_2_H_4_, and CO in the Ground and Excited States of Cu(I)–COF-301

		theor binding enthalpies (kJ/mol)	
gas	exptl *E*_des_ (kJ/mol)	S_0_	S_1_
H_2_	29	42	21
C_2_H_4_	57	121	112
CO	68	126	103

Given this π-back-bonding contribution, the observed trends in *E*_des_ can be explained by orbital overlap between the Cu 3d_π_ orbital and the gas antibonding orbitals, where the π* orbitals in C_2_H_4_ and CO have greater overlap with the Cu 3d orbital than does the σ* of H_2_ in the respective complexes, thereby promoting stronger back-bonding interactions in the former. The computationally estimated binding enthalpies were consistently greater than the experimentally determined *E*_des_ due to assumptions made during modeling, namely, the isolation of the model compound in space which alters geometric restrictions and eliminates interactions of ligands across the entire COF molecule. Nevertheless, the trend in computed binding enthalpies matches well with the experimental *E*_des_, indicating that the electronic structure around the Cu(I) center was treated accurately in the computational modeling.

Furthermore, DFT analyses discovered that UV exposure promotes the Cu(I)–COF-301 model compound into an excited state (S_1_), where the binding enthalpies of H_2_, C_2_H_4_, and CO are consistently lower than in the ground state (Table 2). The S_1_ binding enthalpies were obtained by performing rigid scans on the surface by using the S_1_-optimized geometry for the corresponding COF–gas complexes. Upon excitation to the S_1_ excited state, H_2_ shifts out of plane from the Cu(I) site, and the Cu–H distance increases from 1.64 to 1.98 Å. Likewise, the Cu–C lengthens from 1.81 to 1.92 Å in the S_1_ excited state for the CO bound complex. This bond lengthening corresponds with the reduction in binding enthalpies, as the gas molecules are less tightly bound to the Cu(I) site.

**Figure 6:**
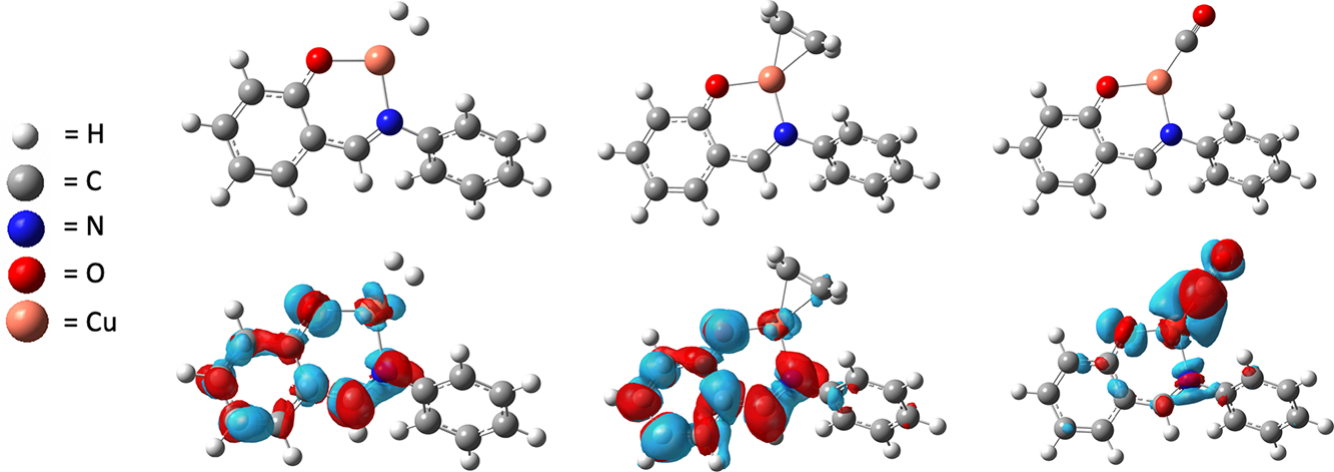
Difference density plots for the Cu(I)–COF-301 model compound bound to H_2_, C_2_H_4_, and CO (left to right). Red and blue contours represents accumulation and depletion of electron density upon excitation.

To gain insight into the nature of the excited states, difference density plots were obtained by subtracting the ground-state electronic density from the Franck–Condon excited-state density. These plots have been used in the past for a variety of systems to consistently provide accurate information about the nature of the excited states.^91,92^ Difference density plots, shown in Figure 6, reveal that promotion to the S_1_ excited state results in reduced electron density in the antibonding orbitals for both the Cu–H_2_ and Cu–CO interactions. Interestingly, the difference density plots did not reveal significant disruption of the Cu–C_2_H_4_ π-back-bonding interactions, and this is reflected in the lesser reduction in S_1_ binding enthalpy compared to H_2_ and CO. This result can be explained by the greater contribution of forward bonding in Cu–C_2_H_4_ complexes compared to Cu–H_2_ and Cu–CO complexes.^67^ Although photothermal heating is responsible for some of the photodriven gas desorption, this DFT analysis suggests that UV light also disrupts π-back-bonding interactions, which would lower the barrier for gas desorption and effectively make photothermal driven desorption more efficient.

## Conclusions

A strategy was presented for loading an imine-based COF containing subnanometer-sized pores with atomically dispersed Cu, after which the material was activated to generate open Cu(I) binding sites. Experimental and computational evidence suggest Cu(I)–COF-301 complex formation with H_2_, C_2_H_4_, and CO can be attributed to π-back-bonding interactions. Cu(I)–COF-301 desorbs H_2_ with the highest reported *E*_des_ of any known COF-based material to date (29 kJ/mol). Furthermore, a light-emitting diode was used to realize the on-demand release of all three gases from the sorbent. Our analyses suggest this response is a function of both localized photothermal effects and the disruption of π-back-bonding in the photoexcited state. Given these results, we contend these framework-based materials are promising for a wide range of gas storage and separation applications, including on-demand H_2_ generation for back-up power systems, olefin separations, and efficient CO scrubbers. Furthermore, this phototriggered gas desorption strategy could be applied to other UV-absorbing frameworks with open metal sites that strongly interact with gases, thereby lowering the temperature of desorption into a more practical range.

## Supporting Information Available

TEM images, nitrogen adsorption isotherms, DRIFTS, XRD, XPS, TPD, thermal imaging data, optimized computational geometries (PDF)
