# Supplementary material for: Phototriggered Desorption of Hydrogen, Ethylene, and Carbon Monoxide from a Cu(I)-Modified Covalent Organic Framework
Source: J Phys Chem C Nanomater Interfaces. 2022 Aug 24;126(35):14801–12. doi: 10.1021/acs.jpcc.2c03194 (PMC9465684; doi:10.1021/acs.jpcc.2c03194)
Supplement: Supplementary file 1 — jp2c03194_si_001.pdf [file jp2c03194_si_001.pdf]

Supporting Information: Photo-Triggered Desorption of Hydrogen, Ethylene, and Carbon Monoxide from a Cu(I)-Modified Covalent Organic Framework

Rachel E. Mow,[†,‡,¶] Lucy J. T. Metzroth,[†,¶] Michael J. Dzara,[‡] Glory A. Russell-Parks,[‡,¶] Justin C. Johnson,[¶] Derek R. Vardon,[¶] Svitlana Pylypenko,[‡,†] Shubham Vyas,[‡,†] Thomas Gennett,[*,‡,¶,†] and Wade A. Braunecker[*,‡,¶]

†*Materials Science Program, Colorado School of Mines, Golden, CO 80401*

‡*Department of Chemistry, Colorado School of Mines, Golden, CO 80401*

¶*National Renewable Energy Laboratory, Golden, CO 80401*

E-mail: tgennett@mines.edu; wade.braunecker@nrel.gov

Contents

Transmission Electron Microscopy S3
Nitrogen Adsorption S3
Infrared Spectroscopy S5
X-Ray Diffraction S6
X-Ray Photoelectron Spectroscopy S7
Temperature Programmed Desorption S8
UV-Vis S13
Thermal Imaging S14
Computational Results S16

Transmission Electron Microscopy

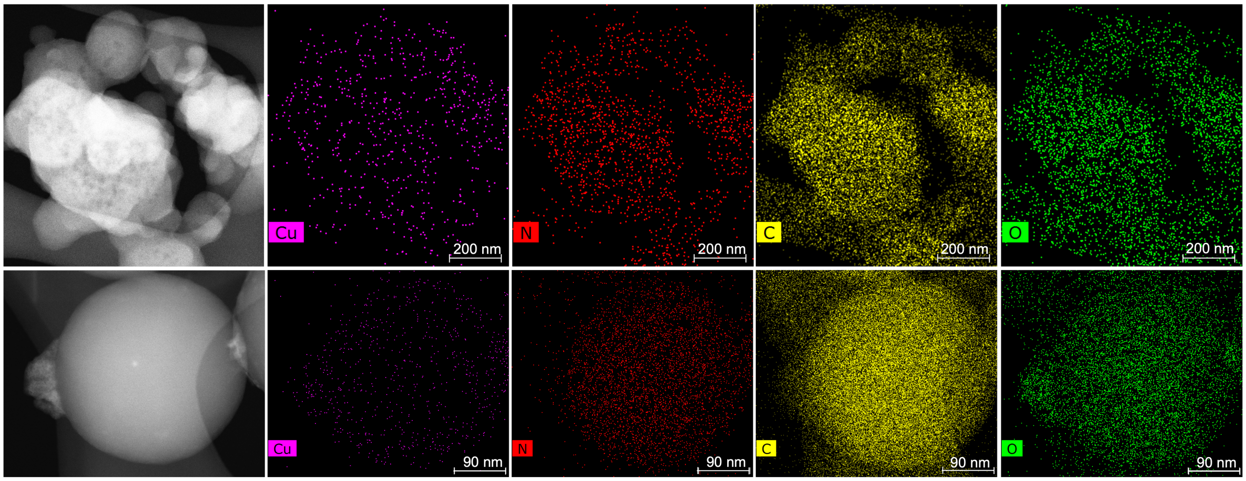

Figure S1: HAADF/STEM images and corresponding EDS maps of Cu (purple), N (red), C (yellow), and O (green) for Cu-loaded COF-301 particles.

Nitrogen Adsorption

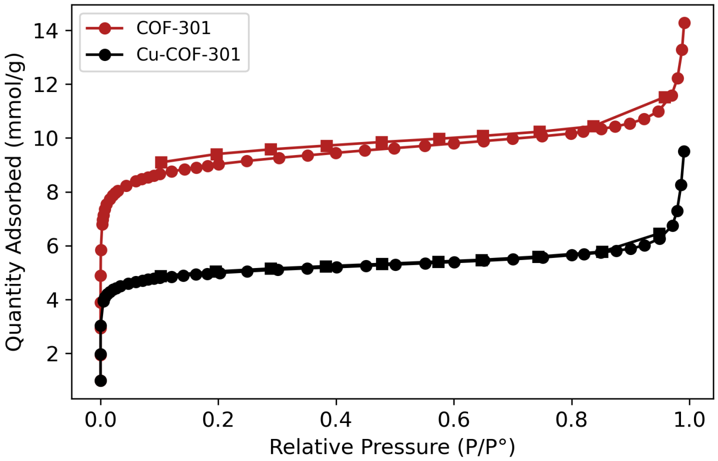

Figure S2: Nitrogen adsorption and surface area decreased from 770 m^2/g to 430 m^2/g when COF-301 was loaded with Cu.

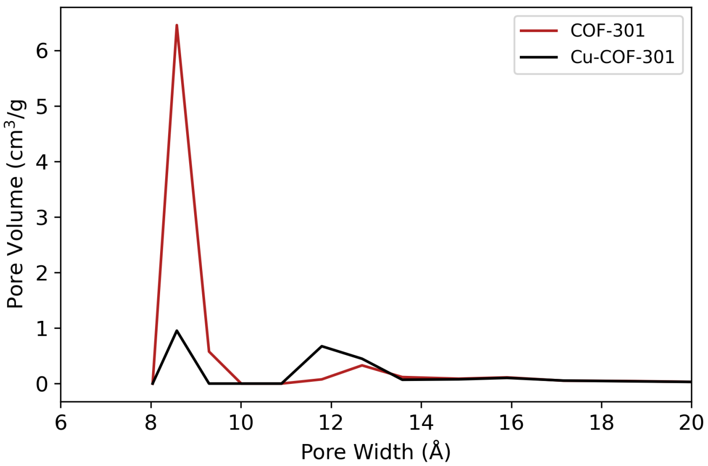

Figure S3: Pore size distributions from DFT slit-pore analysis of BET data. Both the neat and Cu-loaded COF-301 have a pore a 8.6 Å. With Cu-loading, a pore at 12.7 Å shifts to 11.8 Å.

Infrared Spectroscopy

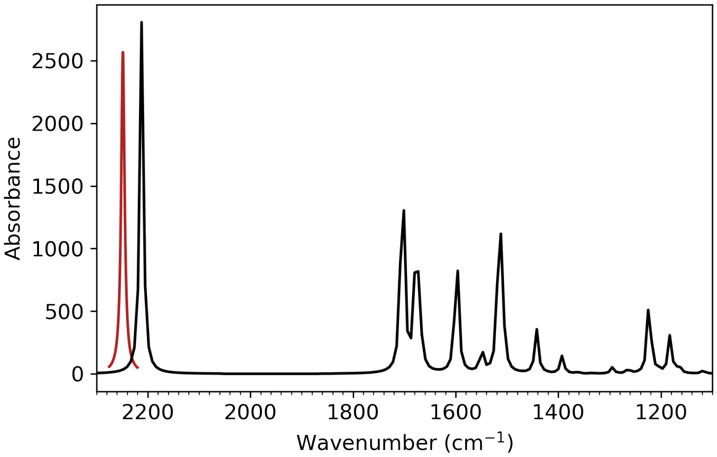

Figure S4: Computed IR spectra for free CO (red) and CO bound to a Cu(2-[(phenylimino)methyl]-phenol) model compound (black), with stretching frequencies of 2248 cm^{-1} and 2212 cm^{-1}, respectively.

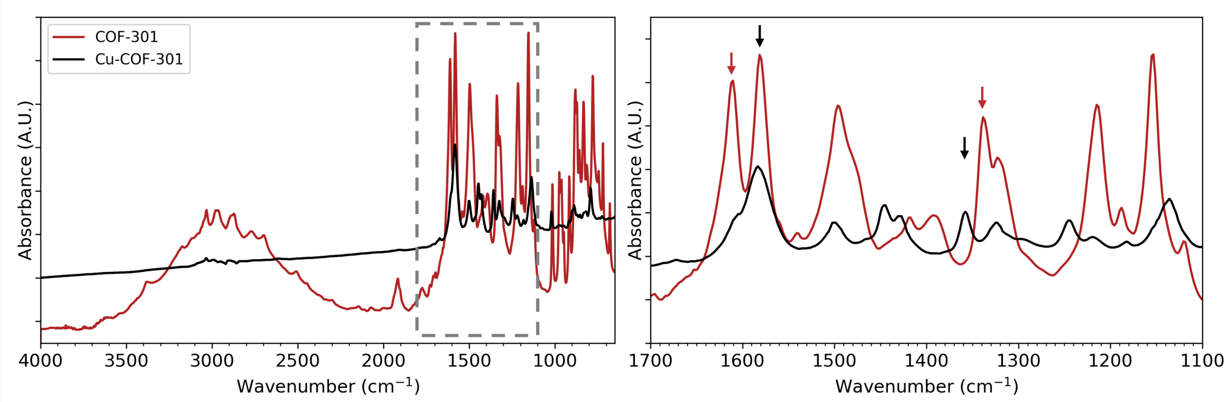

Figure S5: Diffuse reflectance infrared fourier transform spectroscopy (DRIFTS) of COF-301 (red) and Cu(II)-loaded COF-301 (black). A shift in the phenolic C-O band from 1340 to 1360 cm^{-1} and the shift in the C=N stretching band from 1610 cm^{-1} to 1590 cm^{-1} are consistent with the formation of a Schiff base complex

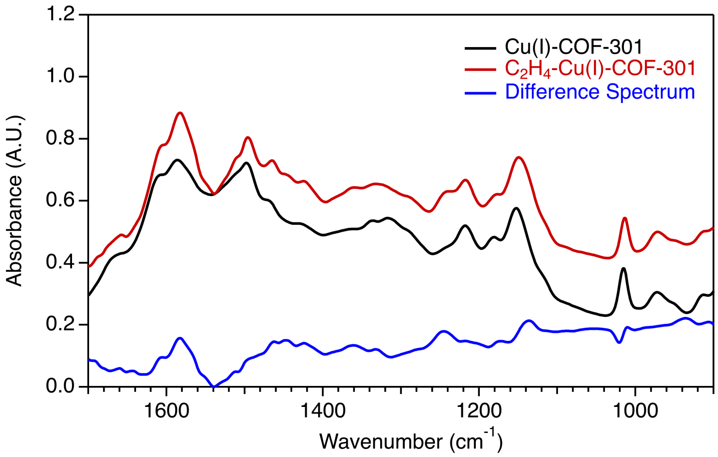

Figure S6: DRIFTS spectra of Cu(I)-COF-301 before and after dosing with ethylene. The appearance of weak bands associated with CH_2 scissoring near 1424 cm^{-1} and 930 cm^{-1}, as well as CH_2 wagging near 1250 cm^{-1}, are consistent with the formation of a stable C_2H_4-Cu(I) complex.

X-Ray Diffraction

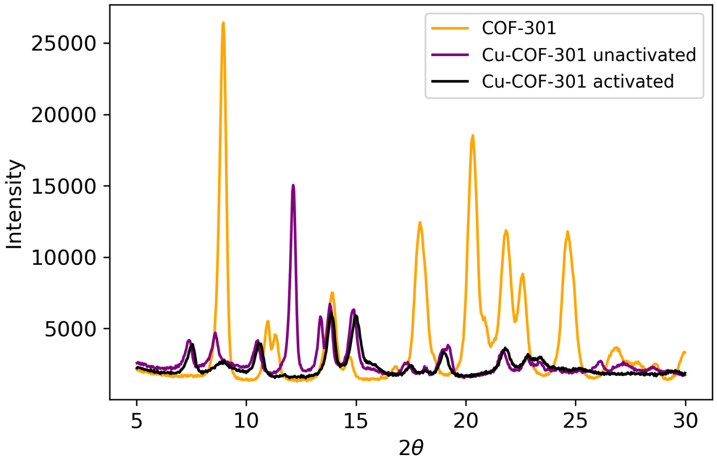

Figure S7: X-ray diffraction of COF-301 (orange), unactivated Cu(II)-COF-301 (purple), and activated Cu(I)-COF-301 (black).

X-Ray Photoelectron Spectroscopy

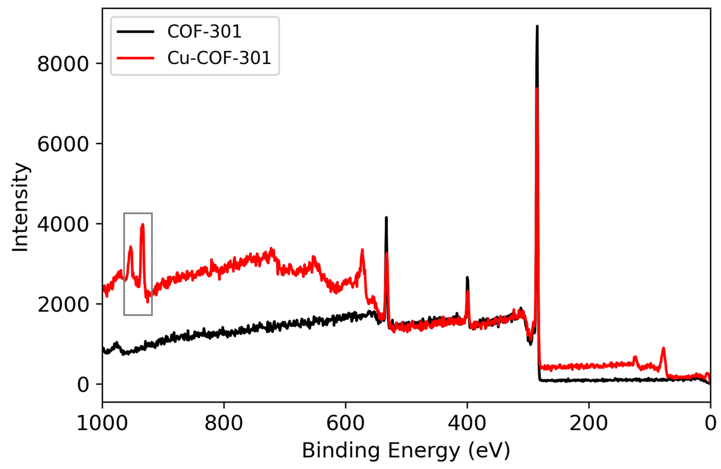

Figure S8: XPS survey of COF-301 (black) and Cu(II)-loaded COF-301 (red), indicative of successful Cu loading (boxed region at 930-960 eV).

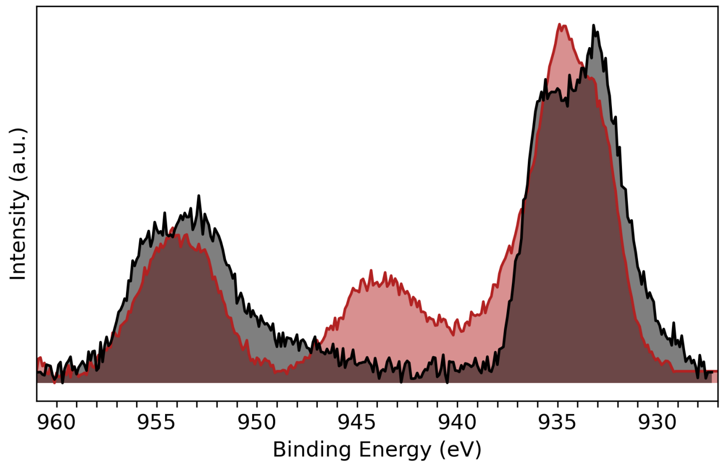

Figure S9: High resolution Cu 2p XPS spectra of Cu-COF-301 pre and post activation

Temperature Programmed Desorption

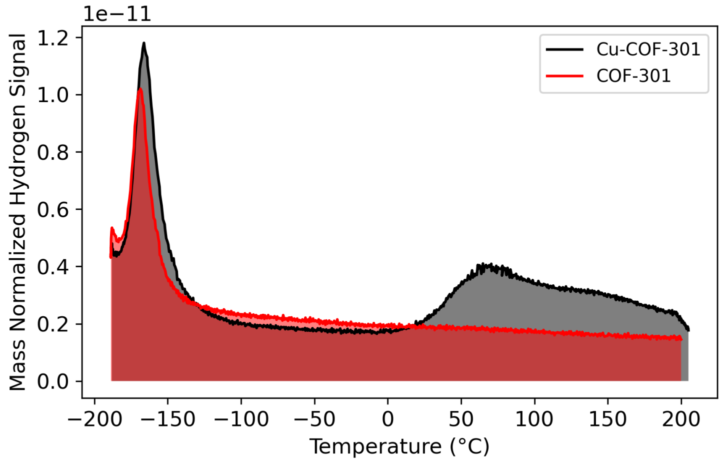

Figure S10: H_2 TPD for COF-301 (red) and Cu-COF-301 (black). H_2 physisorption is observed for both materials at cryogenic temperatures, but H_2 desorption above r.t. is only observed in the Cu-loaded COF.

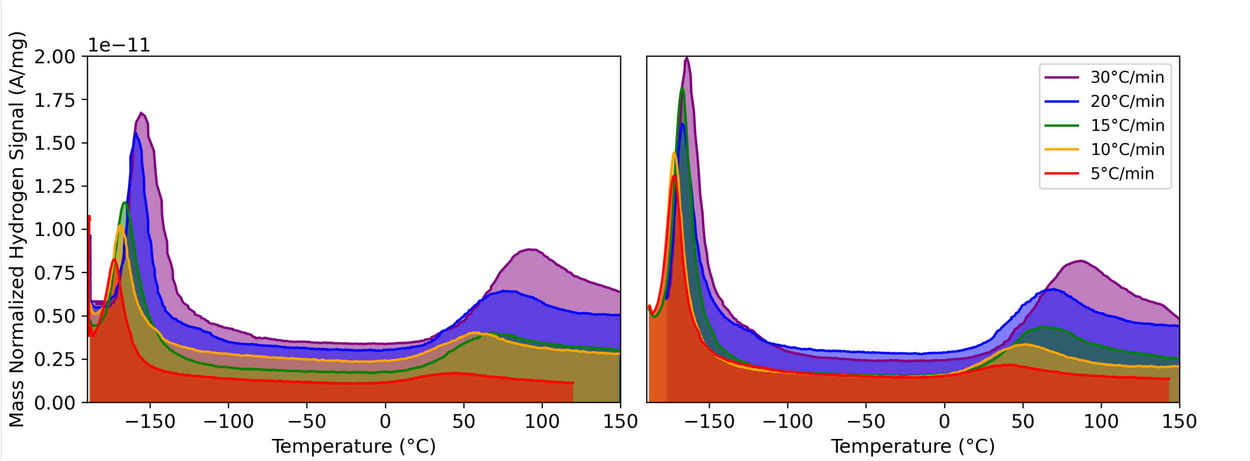

Figure S11: H_2 variable temperature ramp rate TPD experiments performed before (left) and after (right) >20 minutes of exposure to 200 mW/cm^2 irradiation with 385-nm light.

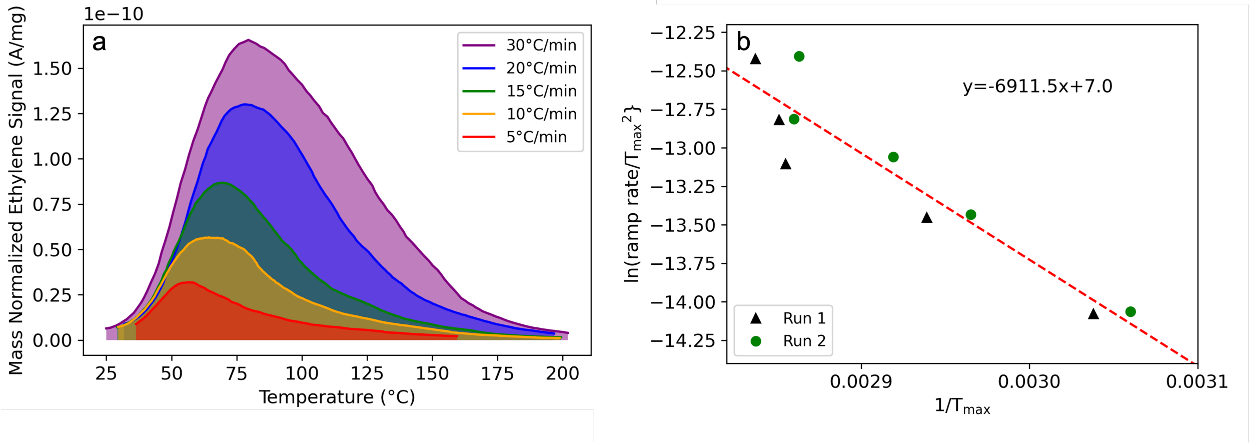

Figure S12: a) C_2H_4 variable temperature ramp rate TPD data. The C_2H_4-Cu(I) complex was sufficiently stable to allow evacuation of the headspace with ultra high vacuum prior to these measurements. b) Kissinger analysis for two TPD cycles was used to calculate an activation energy of 57 kJ/mol.

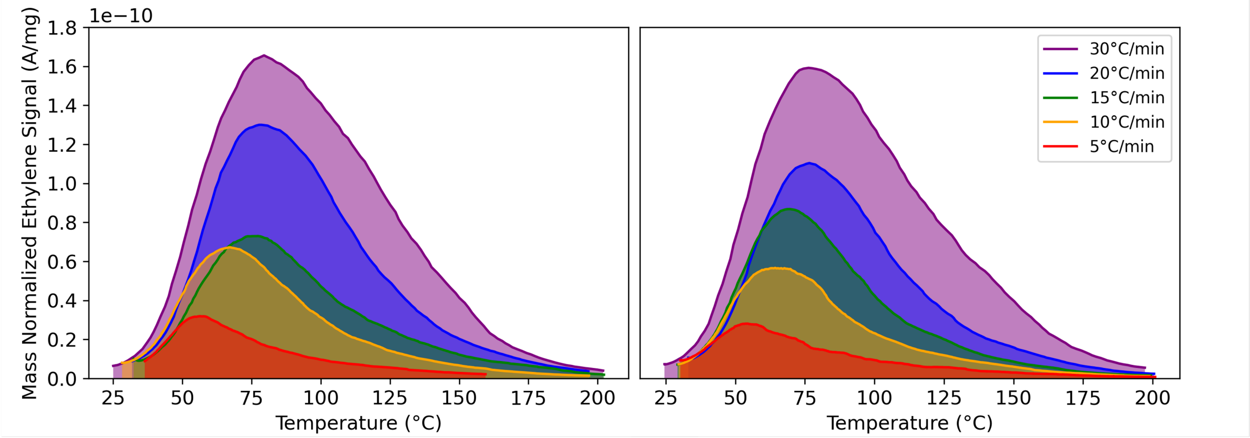

Figure S13: C_2H_4 variable temperature ramp rate TPD experiments performed before (left) and after (right) >20 minutes of exposure to 200 mW/cm^2 irradiation with 385-nm light.

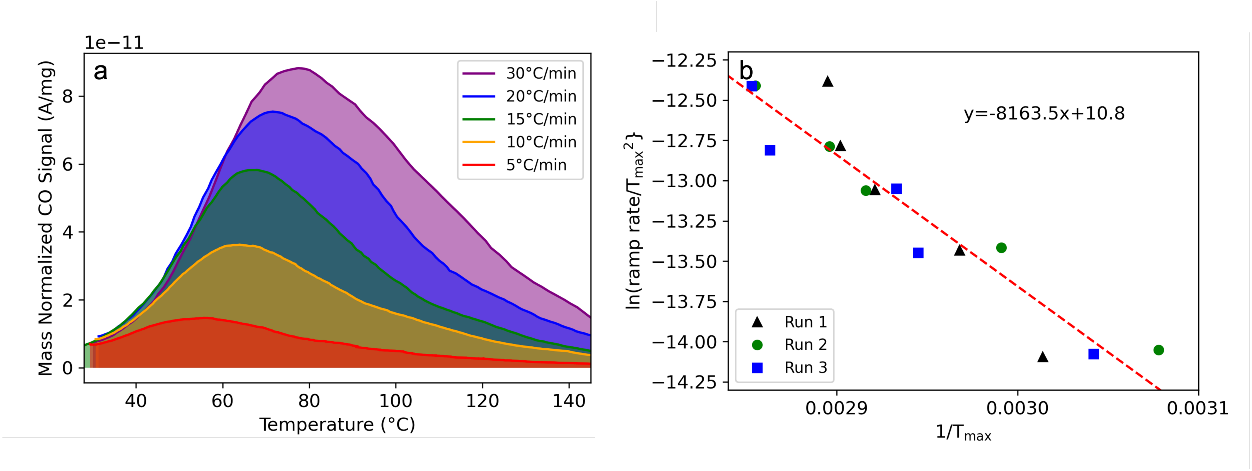

Figure S14: a) CO variable temperature ramp rate TPD experiments. CO-Cu(I) complex was sufficiently stable to allow evacuation of the headspace with ultra high vacuum prior to these measurements. b) Kissinger analysis for three TPD cycles was used to calculate an activation energy of 68 kJ/mol.

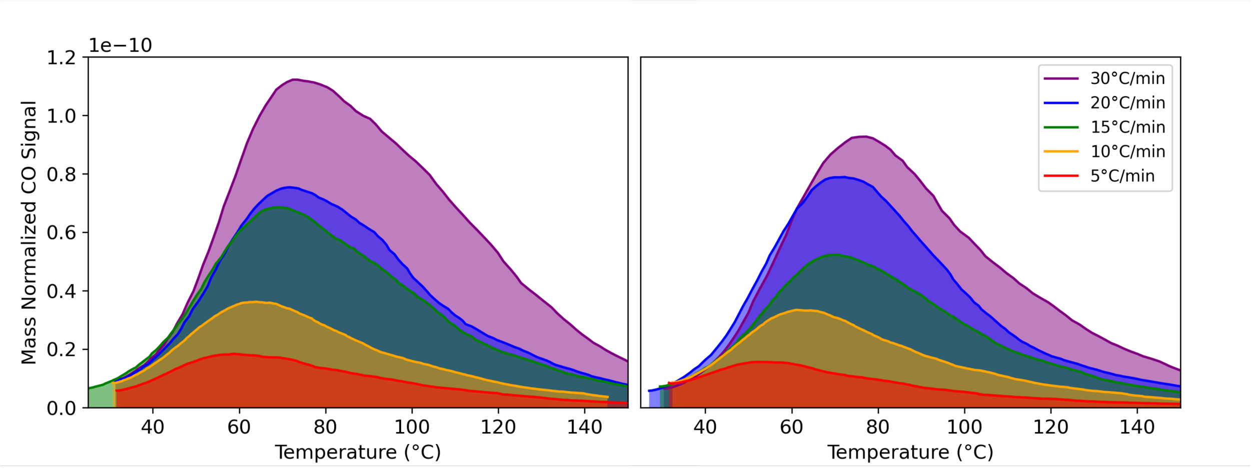

Figure S15: CO variable temperature ramp rate TPD experiments performed before (left) and after (right) >20 minutes of exposure to 200 mW/cm^2 irradiation with 385-nm light

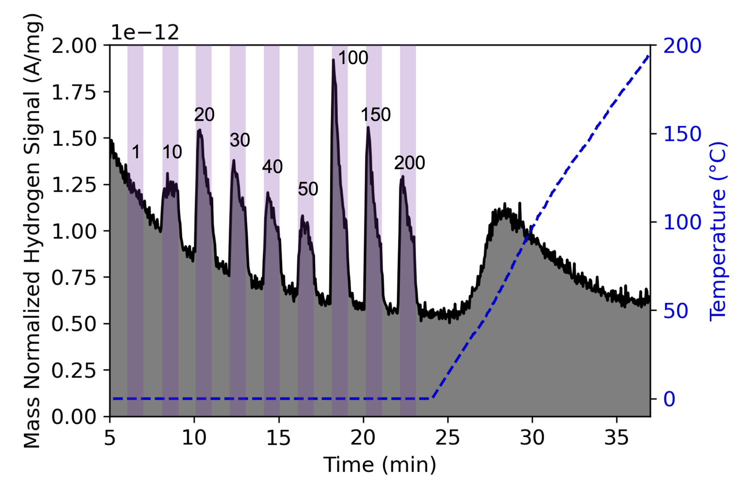

Figure S16: Photo-driven desorption of H_2 from Cu(I)-COF-301. The sample was irradiated for 1 minute with a 385-nm LED with powers ranging from 1-200 mW/cm^2 followed by 1 minute in the dark. The sample was immersed in an ice bath during irradiation because H_2 begin desorbing at room temperature under ultra high vacuum. Purple shading represents sample exposure to LED.

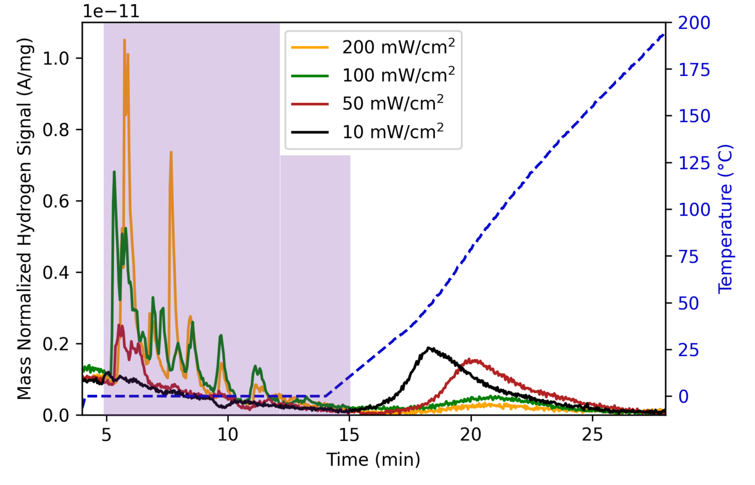

Figure S17: Photo-driven desorption of H_2 from Cu(I)-COF-301 when irradiated with different powers of a 385-nm LED for 10 minutes. Purple shading represents sample exposure to LED.

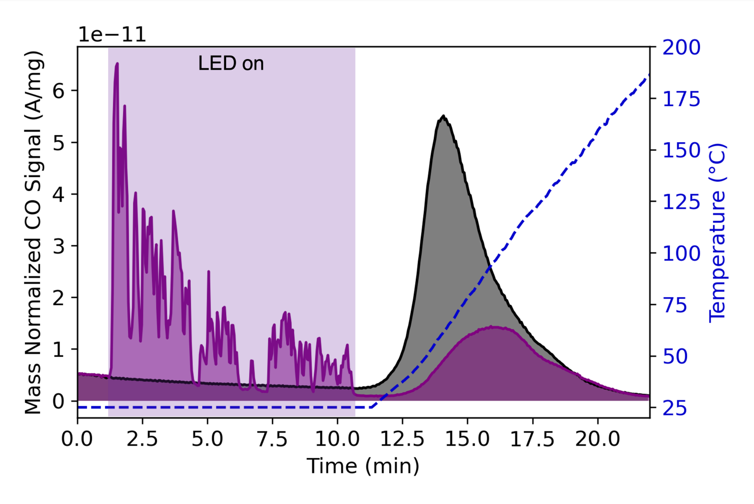

Figure S18: Photo-driven desorption of CO from the Cu(I) site in Cu(I)-COF-301 when irradiated with a 385-nm LED (purple shaded region). Nearly 70% of the CO desorbed from the Cu(I) site with 10 min of 200 mW/cm^2 UV exposure .

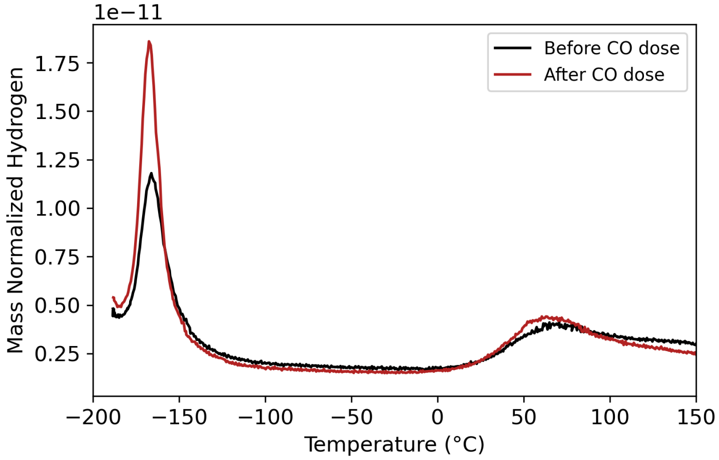

Figure S19: H_2 TPD performed before and after dosing with CO and C_2H_4 showed no reduction in H_2 uptake at the Cu(I) site, indicating CO and C_2H_4 adsorption and desorption were fully reversible

UV-Vis

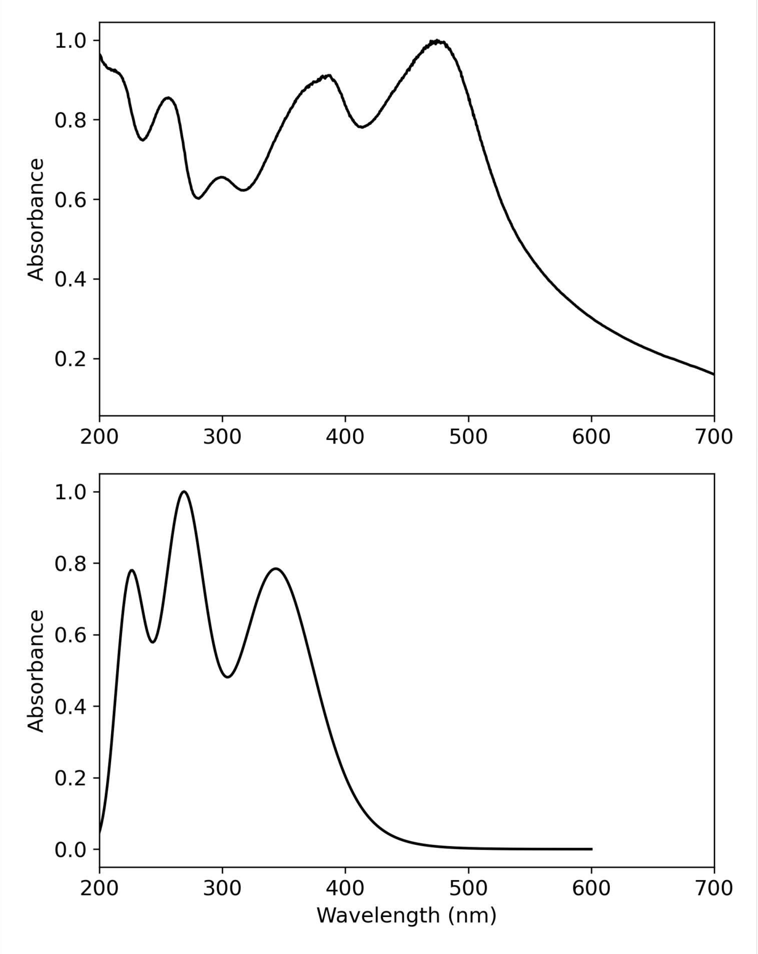

Figure S20: Experimental absorbance spectra of colloidal COF-301 in acetonitrile (top) and computational spectra of model compound (bottom). Colloidal COF-301 was synthesized for absorbance measurements following a previously reported procedure.[1] The absorbance spectrum of the Cu-loaded COF-301 could not be obtained due to irreversible aggregation during Cu-loading.

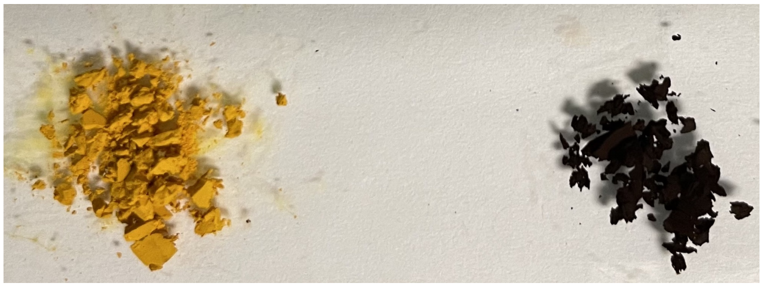

Figure S21: As-synthesized COF-301 is orange (left), and the COF turns black when loaded with Cu and activated (right). This visual observation indicates the Cu-COF-301 will have greater absorption at higher wavelengths than the neat COF.

Thermal Imaging

Table S1: Sample temperatures as a function of irradiance at 385-nm, recorded after 30 s of light exposure in a dry nitrogen environment.

Irradiance @ 385-nm (mW/cm^2)	COF-301 (°C)	Cu-COF-301 (°C)	White enamel hot plate surface (°C)
0	24	24	24
1	24	24	24
10	26	27	24
30	33	34	24
50	38	42	24
100	44	57	25
140	51	70	25
225	66	97	26
325	80	118	27
400	93	137	28

Table S2: Sample temperatures as a function of irradiance at 625 nm, recorded after 30 s of light exposure in a dry nitrogen environment.

Irradiance @ 625 nm (mW/cm^2)	COF-301 (°C)	Cu-COF-301 (°C)	White enamel hot plate surface (°C)
0	24	24	24
1	24	24	24
10	24	27	24
25	25	30	24
40	25	34	24
80	27	44	24
110	29	52	24
185	32	69	24
250	35	83	24
320	39	95	24

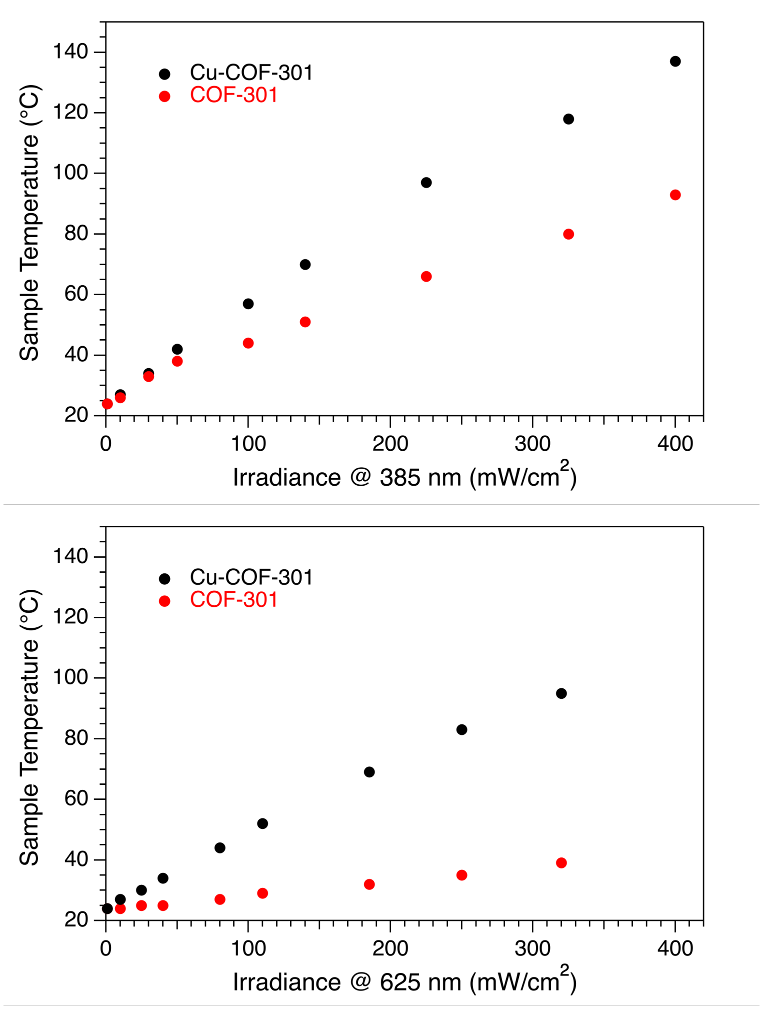

Figure S22: Sample temperature (°C) *vs.* irradiance at 385-nm (top) and 625 nm (bottom). Sample temperatures recorded with a thermal imaging camera in a dry nitrogen atmosphere. Emissivity of 0.85 and 0.90 were estimated for COF-301 and Cu-COF-301, respectively.

Computational Results

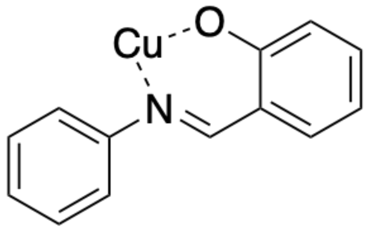

Figure S23: Chemical structure of the linker molecule used for DFT calculations, representative of the Cu(I) binding site in Cu(I)-COF-301

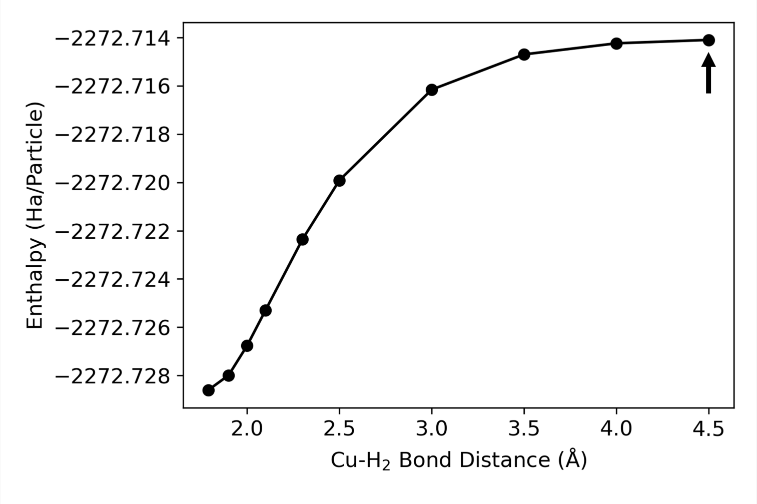

Figure S24: H_2 potential energy surface (PES) calculated from the optimized S_1 structure as H_2 moves away from the Cu center. The arrow represents the transition state that describes H_2 detaching from the linker molecule and thus the term $\Delta H^{\circ}_{linker} + \Delta H^{\circ}_{adsorbate}$ needed to find the binding energy. This technique provides an upper bound to the binding energy of the adsorbate in the excited state.

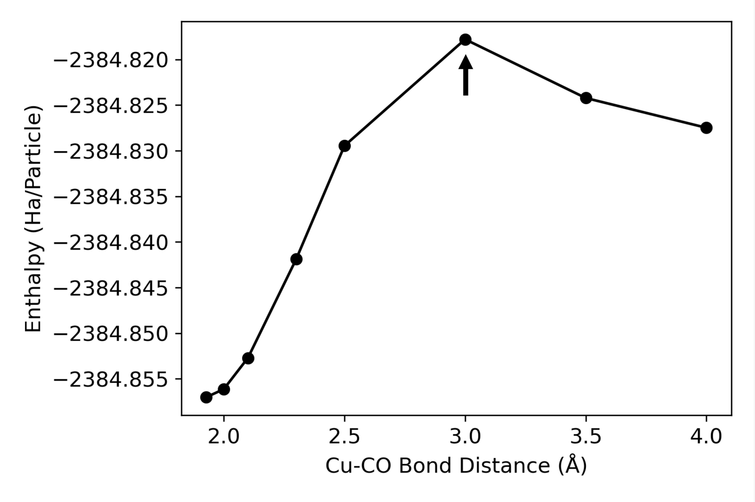

Figure S25: CO PES calculated from the optimized S_1 structure as CO moves away from the Cu center. The arrow represents the transition state where CO detached from the linker molecule.

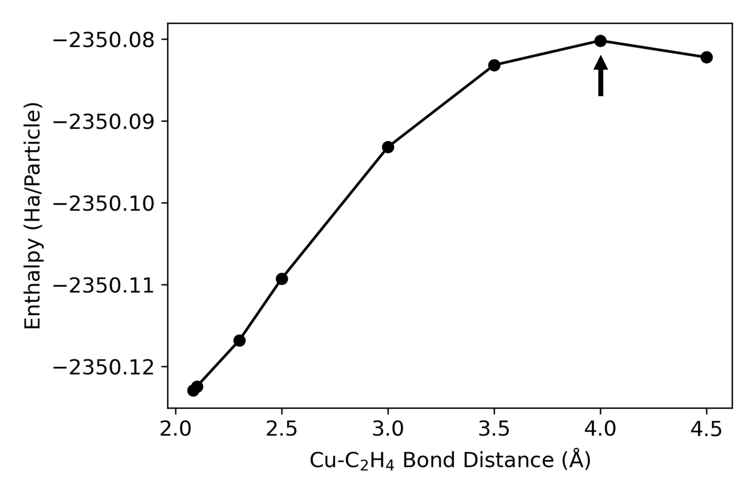

Figure S26: C_2H_4 PES calculated from the optimized S_1 structure as C_2H_4 moves away from the Cu center. The arrow represents the transition state where C_2H_4 detached from the linker molecule.

Table S3: H_2 Ground State CAM-B3LYP Geometries

Atom	X	Y	Z
C	-4.49388	-0.93968	0.096451
C	-3.85978	0.269729	-0.03692
C	-2.43808	0.378963	-0.03522
C	-1.69086	-0.8395	0.109711
C	-2.39051	-2.06853	0.246056
C	-3.75992	-2.13705	0.241966
H	-5.57989	-0.9753	0.090382
H	-4.41757	1.193429	-0.14814
H	-1.80656	-2.97938	0.356756
H	-4.26935	-3.0883	0.347123
C	-0.26391	-0.93007	0.128833
N	0.614952	0.028571	0.040262
C	1.990124	-0.33514	0.03271
C	2.884745	0.368108	0.842732
C	4.233347	0.034375	0.851966
C	4.70979	-0.99614	0.043305
C	3.823566	-1.68797	-0.77589
C	2.470189	-1.36091	-0.78529
H	2.50772	1.167128	1.473464
H	4.915918	0.581282	1.494889
H	5.764499	-1.25086	0.046663
H	4.185575	-2.48101	-1.42279
H	1.788786	-1.88099	-1.45096
O	-1.91112	1.551827	-0.1608
Cu	-0.03077	1.8623	-0.17511
H	0.124877	-1.9465	0.247549
H	0.324004	3.436852	-0.38445
H	1.049685	3.065174	-0.33029

Table S4: CO Ground State CAM-B3LYP Geometries

Atom	X	Y	Z
N	0.452475	-0.38359	0.069819
C	-0.52119	-1.24867	0.104398
C	1.782228	-0.8906	0.049691
C	-1.92729	-0.9988	0.083354
H	-0.24401	-2.30564	0.171414
Cu	0.063562	1.548292	-0.06309
C	2.733696	-0.34308	0.913223
C	2.162673	-1.90347	-0.83395
C	-2.77143	-2.13816	0.154191
C	-2.51467	0.306555	-0.00289
O	-1.83754	1.405332	-0.06701
C	4.039251	-0.8185	0.909046
H	2.434466	0.445906	1.595981
C	3.473562	-2.37263	-0.83735
H	1.439249	-2.3017	-1.53829
C	-4.13887	-2.03428	0.140575
H	-2.30487	-3.11831	0.220977
C	-3.93757	0.378336	-0.0163
C	4.415708	-1.83615	0.034277
H	4.766377	-0.3923	1.593077
H	3.759192	-3.15411	-1.53456
C	-4.71718	-0.74921	0.053119
H	-4.76411	-2.91833	0.195097
H	-4.37589	1.36832	-0.08293
H	5.437431	-2.20126	0.027729
H	-5.79912	-0.64994	0.040779
C	1.109308	3.022243	-0.18982
O	1.776455	3.940981	-0.27271

Table S5: C_2H_4 Ground State CAM-B3LYP Geometries

Atom	X	Y	Z
C	-4.64827	-0.99458	0.068171
C	-3.93107	0.174239	0.011879
C	-2.50513	0.181125	0.014736
C	-1.84893	-1.09279	0.080106
C	-2.62988	-2.27674	0.136455
C	-4.0013	-2.24794	0.131479
H	-5.73415	-0.95336	0.063576
H	-4.42325	1.139734	-0.03728
H	-2.11036	-3.23106	0.185747
H	-4.57732	-3.16543	0.175134
C	-0.43067	-1.2678	0.095089
N	0.496373	-0.35373	0.081237
C	1.847478	-0.79912	0.055085
C	2.747295	-0.32053	1.009959
C	4.070895	-0.74373	0.997802
C	4.516316	-1.63844	0.026141
C	3.624251	-2.1075	-0.93273
C	2.295478	-1.69102	-0.9219
H	2.392647	0.369898	1.768196
H	4.758112	-0.37477	1.752876
H	5.551707	-1.96281	0.015359
H	3.962268	-2.79623	-1.70078
H	1.606333	-2.03726	-1.6858
O	-1.89026	1.314391	-0.04218
C	0.005991	1.549302	-0.05735
H	-0.09759	-2.31004	0.137836
C	1.552586	2.839709	-0.20545
H	2.102173	2.574021	-1.10493
H	2.116803	2.790782	0.721892
C	0.354474	3.517081	-0.27269
H	-0.08591	3.793028	-1.22712
H	-0.06887	4.002931	0.602395

Table S6: H_2 Excited State CAM-B3LYP Geometries

Atom	X	Y	Z
C	-4.56427	-0.53973	-0.1245
C	-3.71123	0.521675	-0.3887
C	-2.3184	0.380948	-0.30152
C	-1.75893	-0.86183	0.162777
C	-2.6689	-1.91835	0.431219
C	-4.03261	-1.77223	0.283936
H	-5.6365	-0.41233	-0.23189
H	-4.09341	1.481414	-0.7205
H	-2.26346	-2.8642	0.780234
H	-4.69184	-2.60611	0.503029
C	-0.38056	-1.06447	0.404864
N	0.586598	-0.10034	0.445542
C	1.917284	-0.40668	0.193709
C	2.916842	0.461534	0.682186
C	4.258415	0.223209	0.420549
C	4.64528	-0.90132	-0.30688
C	3.666735	-1.7795	-0.77581
C	2.320809	-1.5411	-0.54046
H	2.619625	1.300057	1.30793
H	5.009005	0.905261	0.808198
H	5.695086	-1.09744	-0.4976
H	3.958142	-2.65671	-1.34585
H	1.566179	-2.20905	-0.94066
O	-1.56101	1.404211	-0.6742
Cu	0.132694	1.659667	0.046385
H	-0.07215	-2.06172	0.714273
H	0.074551	3.367729	-0.4199
H	0.625763	3.378442	0.12415

Table S7: CO Excited State CAM-B3LYP Geometries

Atom	X	Y	Z
N	0.328045	-0.36505	0.108875
C	-0.66095	-1.20324	0.099651
C	1.663928	-0.85373	0.086047
C	-2.05942	-0.89607	0.057366
H	-0.40778	-2.26556	0.144996
Cu	0.043932	1.553227	-0.03703
C	2.541053	-0.48841	1.107173
C	2.100766	-1.67105	-0.9584
C	-2.97203	-1.9758	0.085136
C	-2.55495	0.439022	0.009049
O	-1.78247	1.488205	-0.02206
C	3.849654	-0.95754	1.089257
H	2.190383	0.157849	1.904474
C	3.410044	-2.13812	-0.96602
H	1.420053	-1.91737	-1.76711
C	-4.33162	-1.77153	0.066204
H	-2.57529	-2.98703	0.123336
C	-3.95715	0.625609	-0.00185
C	4.287009	-1.78253	0.056214
H	4.530282	-0.67393	1.885207
H	3.748986	-2.77062	-1.78009
C	-4.81561	-0.45149	0.022627
H	-5.01816	-2.61007	0.087034
H	-4.32571	1.644669	-0.03502
H	5.3108	-2.14167	0.043379
H	-5.88701	-0.27399	0.009033
C	1.755547	2.396014	-0.31043
O	2.770662	2.977116	-0.3474

Table S8: C_2H_4 Excited State CAM-B3LYP Geometries

Atom	X	Y	Z
C	-4.58379	-0.92858	0.002275
C	-3.75754	0.09425	-0.44742
C	-2.35038	-0.03072	-0.42842
C	-1.75049	-1.21636	0.169262
C	-2.62235	-2.21755	0.620488
C	-4.00909	-2.09017	0.532921
H	-5.6622	-0.82674	-0.05573
H	-4.16348	0.997016	-0.89216
H	-2.19772	-3.11298	1.066191
H	-4.64213	-2.89259	0.898031
C	-0.34001	-1.38661	0.307715
N	0.580347	-0.40005	0.252604
C	1.919069	-0.74587	0.094012
C	2.917597	0.115875	0.590838
C	4.261825	-0.18653	0.437848
C	4.654871	-1.36005	-0.20677
C	3.677229	-2.22346	-0.70003
C	2.328683	-1.92826	-0.55684
H	2.610743	1.006243	1.129556
H	5.010985	0.49007	0.838147
H	5.707135	-1.5991	-0.32028
H	3.969636	-3.13328	-1.21596
H	1.581256	-2.58719	-0.98467
O	-1.61857	0.886392	-0.98622
Cu	0.00841	1.40903	-0.03579
H	0.005058	-2.38238	0.582254
C	0.773964	3.211581	0.674513
H	1.815533	3.246478	0.36785
H	0.585746	3.212828	1.744622
C	-0.2354	3.426541	-0.22711
H	-0.02432	3.631136	-1.27342
H	-1.25916	3.593267	0.095323

Table S9: Time Dependent Density Functional Theory values show the first twenty excited states of the linker molecule representing COF-301. These values were used to generate the UV-Vis spectrum benchmarking the linker molecule to the experimental COF-301 structure.

	Energy (eV)	Wavelength (nm)	Oscillator Strength
Excited State 1:	3.0509	406.39	.0057
Excited State 2:	3.5034	353.89	.1088
Excited State 3:	3.6073	343.70	.1406
Excited State 4:	3.6946	335.58	.0329
Excited State 5:	3.9294	315.53	.0031
Excited State 6:	3.9811	311.43	.0386
Excited State 7:	4.1159	301.23	.0164
Excited State 8:	4.5090	274.97	.0031
Excited State 9:	4.5908	270.07	.3046
Excited State 10:	4.7364	261.77	0.0634
Excited State 11:	5.0514	245.44	0.0036
Excited State 12:	5.1713	239.75	0.0032
Excited State 13:	5.1965	238.59	0.0023
Excited State 14:	5.2297	237.08	0.0207
Excited State 15:	5.3823	230.35	0.0331
Excited State 16:	5.4347	228.13	0.0076
Excited State 17:	5.4776	226.35	0.1411
Excited State 18:	5.5791	222.23	0.0588
Excited State 19:	5.6422	219.74	0.0403
Excited State 20:	5.7032	217.39	0.0041

Table S10: Time Dependent Density Functional Theory values show the first twenty excited states of H_2 bound to the Cu(I) binding site in the linker molecule.

	Energy (eV)	Wavelength (nm)	Oscillator Strength
Excited State 1:	3.6165	342.83	0.2796
Excited State 2:	3.9638	312.79	0.0033
Excited State 3:	4.7501	261.02	0.3422
Excited State 4:	5.0592	245.07	0.0056
Excited State 5:	5.2523	236.06	0.0022
Excited State 6:	5.2938	234.20	0.0642
Excited State 7:	5.3765	230.61	0.0939
Excited State 8:	5.4297	228.34	0.0116
Excited State 9:	5.4485	227.56	0.0614
Excited State 10:	5.5481	223.47	0.1115
Excited State 11:	5.5841	222.03	0.0596
Excited State 12:	5.6036	221.26	0.0058
Excited State 13:	5.7461	215.77	0.0497
Excited State 14:	5.7700	214.88	0.0202
Excited State 15:	5.8219	212.96	0.0991
Excited State 16:	5.8322	212.58	0.0299
Excited State 17:	5.9481	208.44	0.0013
Excited State 18:	5.9836	207.21	0.0507
Excited State 19:	6.0339	205.48	0.0084
Excited State 20:	6.1065	203.04	0.0673

Table S11: Time Dependent Density Functional Theory values show the first twenty excited states of CO bound to the Cu(I) binding site in the linker molecule.

	Energy (eV)	Wavelength (nm)	Oscillator Strength
Excited State 1	3.5713	347.17	0.0385
Excited State 2	3.6478	339.89	0.2297
Excited State 3	4.0525	305.95	0.0132
Excited State 4	4.0916	303.02	0.0142
Excited State 5	4.4853	276.43	0.0043
Excited State 6	4.6649	265.78	0.0165
Excited State 7	4.6942	264.12	0.0541
Excited State 8	4.7739	259.71	0.3840
Excited State 9	4.9865	248.64	0.0183
Excited State 10	5.0330	246.34	0.1829
Excited State 11	5.0810	244.01	0.0163
Excited State 12	5.1223	242.05	0.0046
Excited State 13	5.3182	233.13	0.0052
Excited State 14	5.4028	229.48	0.0456
Excited State 15	5.4342	228.16	0.0375
Excited State 16	5.4763	226.40	0.0110
Excited State 17	5.5838	222.04	0.0324
Excited State 18	5.6768	218.40	0.0657
Excited State 19	5.7000	217.52	0.1158
Excited State 20	5.7675	214.97	0.0461

Table S12: Time Dependent Density Functional Theory values show the first twenty excited states of C_2H_4 bound to the Cu(I) binding site in the linker molecule.

	Energy (eV)	Wavelength (nm)	Oscillator Strength
Excited State 1:	3.6305	341.51	0.2391
Excited State 2:	4.0757	304.20	0.0050
Excited State 3:	4.8260	256.91	0.3128
Excited State 4:	4.8458	255.86	0.0033
Excited State 5:	5.1050	242.87	0.0124
Excited State 6:	5.1401	241.21	0.0473
Excited State 7:	5.1942	238.70	0.0078
Excited State 8:	5.3780	230.54	0.1321
Excited State 9:	5.4343	228.15	0.1523
Excited State 10:	5.4984	225.49	0.0693
Excited State 11:	5.5147	224.83	0.0316
Excited State 12:	5.5634	222.86	0.0541
Excited State 13:	5.6314	220.16	0.0230
Excited State 14:	5.6737	218.52	0.2106
Excited State 15:	5.6950	217.71	0.0356
Excited State 16:	5.7612	215.21	0.0117
Excited State 17:	5.7772	214.61	0.0602
Excited State 18:	5.8647	211.41	0.0560
Excited State 19:	5.8996	210.16	0.0025
Excited State 20:	5.9411	208.69	0.0060

References

(1) Mow, R. E.; Lipton, A. S.; Shulda, S.; Gaulding, E. A.; Gennett, T.; Braunecker, W. A. Colloidal three-dimensional covalent organic frameworks and their application as porous liquids. *J. Mater. Chem. A* **2020**, *8*, 23455–23462.
